# TRAF4/6 Is Needed for CD44 Cleavage and Migration via RAC1 Activation

**DOI:** 10.3390/cancers13051021

**Published:** 2021-03-01

**Authors:** Constantinos Kolliopoulos, Athanasios Chatzopoulos, Spyros S. Skandalis, Carl-Henrik Heldin, Paraskevi Heldin

**Affiliations:** 1 Department of Medical Biochemistry and Microbiology, Uppsala University, Box 582, SE-751 23 Uppsala, Sweden; konstantinos.kolliopoulos@imbim.uu.se (C.K.); athanasios.chatzoapoulos@imbim.uu.se (A.C.); c-h.heldin@imbim.uu.se (C.-H.H.); 2 Laboratory of Biochemistry, Department of Chemistry, University of Patras, 26110 Patras, Greece; skandalis@upatras.gr

**Keywords:** CD44, hyaluronan, TRAF, RAC1, migration, stemness

## Abstract

**Simple Summary:**

Tumor cells receive signals from the surrounding extracellular matrix that affect their growth and survival. An important component of the extracellular matrix is the large polysaccharide hyaluronan, which binds and activates certain receptors at the cell surface, including CD44. Activation of CD44 initiates several signaling pathways; one of them involves the cleavage of CD44 by proteases, leading to the release of the intracellular domain of CD44, which after translocation to the nucleus affects the transcription of certain genes. In the present report, we elucidate the mechanism by which CD44 is cleaved, and show that this occurs at an increased rate in stem-like tumor cells grown in spheres. We also show that CD44 cleavage promotes the migration of tumor cells. Since the mechanism we have elucidated promotes tumorigenesis, it is possible that inhibition of this pathway may be beneficial in the treatment of tumor patients.

**Abstract:**

The hyaluronan receptor CD44 can undergo proteolytic cleavage in two steps, leading to the release of its intracellular domain; this domain is translocated to the nucleus, where it affects the transcription of target genes. We report that CD44 cleavage in A549 lung cancer cells and other cells is promoted by transforming growth factor-beta (TGFβ) in a manner that is dependent on ubiquitin ligase tumor necrosis factor receptor-associated factor 4 or 6 (TRAF4 or TRAF6, respectively). Stem-like A549 cells grown in spheres displayed increased TRAF4-dependent expression of CD44 variant isoforms, CD44 cleavage, and hyaluronan synthesis. Mechanistically, TRAF4 activated the small GTPase RAC1. CD44-dependent migration of A549 cells was inhibited by siRNA-mediated knockdown of TRAF4, which was rescued by the transfection of a constitutively active RAC1 mutant. Our findings support the notion that TRAF4/6 mediates pro-tumorigenic effects of CD44, and suggests that inhibitors of CD44 signaling via TRAF4/6 and RAC1 may be beneficial in the treatment of tumor patients.

## 1. Introduction

Hyaluronan, a prominent constituent of the extracellular matrix, is synthesized by three hyaluronan synthase isoforms, i.e., HAS1, HAS2, and HAS3, and is catabolized by hyaluronidases, including HYAL1, HYAL2, and TMEM2. In addition to its structural role in the extracellular matrix, hyaluronan exerts signaling effects via binding to specific cell surface receptors, such as CD44 [1,2,3], which is a transmembrane receptor that mediates signals affecting cell proliferation and migration [4,5]. The human CD44 gene is composed of 19 exons, i.e., 10 “constant exons” encoding the standard CD44 molecule (CD44s) and nine “variable exons”, which in different combinations can be inserted in the membrane-proximal stem region through alternative splicing to generate CD44v isoforms [6]. Certain CD44v isoforms bind chondroitin sulfate and heparan sulfate in addition to hyaluronan. Importantly, expression of both CD44s and CD44v isoforms has been linked to tumor progression [7,8,9].

CD44 isoforms interact with certain adaptor proteins and receptors [3,10,11], and consist of different functional domains, including the N-terminal extracellular domain that binds various ligands; the stem domain, which possesses proteolytic cleavage sites for matrix metalloproteases (MMPs) and ADAM-like proteases [12,13]; a transmembrane domain; and an intracellular domain. The MMP-mediated cleavage of the CD44 ectodomain allows the release of cancer cells bound to hyaluronan, promoting their migration through hyaluronan-containing extracellular matrix (ECM) and facilitating cancer cell dissemination [12,13]. The resulting transmembrane and intracellular domain (CD44 TM-ICD) is subject to a second cleavage in the transmembrane domain by presenilin 1, the catalytic subunit of γ-secretase, releasing the CD44 cytoplasmic region (CD44 ICD) [14]. CD44′s intracellular domain (ICD) possesses signaling functions through interactions with key regulatory molecules, such as Ezrin, radixin and moesin (ERM) proteins and small Rho GTPases [5,15,16,17]. Furthermore, the CD44 ICD regulates the transcription of target genes by forming complexes with cAMP response element-binding protein (CREB)-binding protein (CBP)/p300 or STAT3 [9,18,19,20,21].

Transforming growth factor β (TGFβ) exerts its cellular effects by forming a complex of TGFβ type I (TβRI) and type II (TβRII) receptors [22]. Within the complex, TβRII phosphorylates and activates TβRI, promoting the phosphorylation of Smad molecules that are translocated to the nucleus, where they regulate the transcription of certain genes. In addition, TGFβ activates several non-Smad signaling pathways, including MAP kinase, phosphatidyl-inositol-3′-kinase (PI3-kinase), the tyrosine kinase Src, and similar to CD44, release the intracellular domain of TβRI (TβRI-ICD) [23], which acts as a transcription factor in the nucleus [24]. Some of the non-Smad pathways, including p38 and JNK MAP kinases [25,26], PI3-kinase [27], and release of TβRI-ICD [23], are activated through mechanisms involving ubiquitin ligase tumor necrosis factor receptor associated factor (TRAF) 6.

The TRAF family consists of seven members (TRAF1–TRAF7), which play important roles in signaling events and are implicated in many diseases, including inflammation and cancer [28,29]. Their main characteristics are a RING domain that has E3 ligase activity, followed by a zinc-finger motif and two TRAF domains that function as scaffolds in signaling via Toll-like receptors and receptors for TNF, TGFβ, and cytokines [30,31].

We present here a TRAF4/6-dependent mechanism for how CD44 is cleaved, and demonstrate that the cleavage occurs at increased rate in stem-like A549 lung cancer cells grown in spheres. Moreover, we show that migration of A549 cells is dependent on TRAF4 and its downstream effector RAC1.

## 2. Results

### 2.1. Cleavage of CD44 in Response to External Stimuli

**Figure 1:**
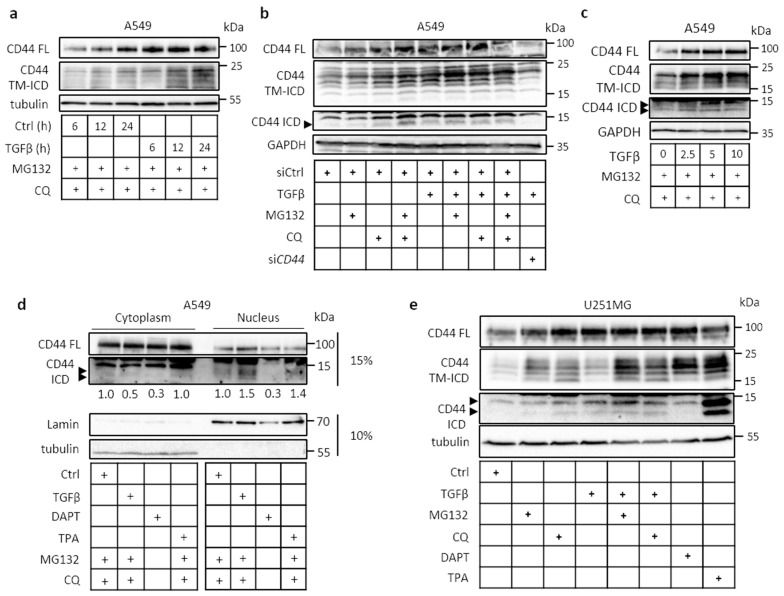
Transforming growth factor β (TGFβ) induces ectodomain and intramembranous cleavage of CD44 in a dose- and time-dependent manners. (**a**,**b**) Immunoblot analysis of full-length (FL) and cleavage products (transmembrane and intracellular domain (TM-ICD) and ICD) of CD44 in A549 cells after treatment or no treatment with TGFβ (5 ng/mL) for the indicated time periods; knockdown of *CD44* by siRNA was performed to confirm the specificity of observed bands (**b**). (**c**) Immunoblotting of FL CD44, as well as the TM-ICD and ICD of CD44 after 24 h of treatment with different concentrations of TGFβ. (**d**), A549 cells treated or not with TGFβ (5 ng/ml), DAPT (40 μM), MG132 and CQ for 24 h, or TPA (80 nM) for 1 h, were subjected to nuclear/cytoplasmic fractionation and subjected to SDS gel electrophoresis in 15% or 10% polyacrylamide gels, followed by immunoblotting for FL, ICD of CD44, lamin, and tubulin. Quantification of CD44-ICD bands was performed via ImageJ after normalizing to CD44 FL (**e**), Immunoblot analysis of FL CD44, as well as the TM-ICD and ICD of CD44 in U251MG cells treated or not treated with TGFβ for 24 h, or 12-O-tetradecanoylphorbol-13-acetate (TPA) (80 nM) for 1 h. The cultures were treated with the γ-secretase inhibitor DAPT (40 μM)), the proteasomal inhibitor MG132, and the lysosomal inhibitor chloroquine (CQ) for 24 h; tubulin or glyceraldehyde 3-phosphate dehydrogenase (GAPDH) were used as loading controls in the same immunoblots. Arrowheads indicate CD44 ICD-like products.

TGFβ promoted the cleavage of CD44 and formation of the CD44 TM-ICD domain in a time-dependent manner in the lung adenocarcinoma A549 cell line (Figure 1a), as did several other growth factors (Figure S1a). We also investigated the effect of proteasomal (MG132) and lysosomal (chloroquine (CQ)) inhibitors on the accumulation of CD44 cleavage products in untreated and TGFβ-treated cells. In TGFβ stimulated cultures, the CD44 TM-ICD and CD44 ICD products increased, indicating that they are unstable and subject to degradation in lysosomes and proteasomes (Figure 1a,b). The CD44 TM-ICD and CD44 ICD products were absent in CD44-depleted cells (Figure 1b), and TGFβ promoted CD44 cleavage in a concentration-dependent manner (Figure 1c). Following CD44 cleavage, translocation of CD44 ICD to the nucleus was detected in untreated cells consistent with its role in transcription regulation; TGFβ and TPA stimulation further enhanced its translocation (Figure 1d). The constitutive, TGFβ- and TPA-mediated generation of CD44 ICD was abolished after incubation with the γ-secretase inhibitor DAPT. Certain smaller CD44 ICD-like bands were detected; it is possible that these are generated due to less specific proteolytic cleavage performed by presenilin 1/γ-secretase activity and/or because CD44 ICD is highly susceptible to degradation (Figure 1c,d,e). Similarly, in TGFβ-treated glioblastoma U251MG cell cultures, the CD44 TM-ICD and CD44 ICD products were clearly detected in TGFβ-stimulated cultures after the inhibition of lysosomal or proteasomal degradation (Figure 1e). In U251MG cells treated with the γ-secretase inhibitor N-[N-3,5-Difluorophenacetyl)-L-alanyl]-S-phenylglycine t-butyl ester (DAPT), the induction of CD44 TM-ICD product was increased—whereas, as expected, no CD44 ICD was detected (Figure 1e). Moreover, as in A549 cells, the proteolytic cleavage products of CD44 in U251MG cells were sensitive to lysosomal and proteasomal degradation. TPA (12-O-tetradecanoylphorbol-13-acetate), a protein kinase C activator that has been demonstrated to promote the proteolytic cleavage of several cell surface receptors [32], was used as a positive control. Since both A549 and U251MG cells synthesize hyaluronan, we investigated the effect of exogenous hyaluronan on CD44 cleavage; both fragmented and high-molecular-mass hyaluronan promoted CD44 cleavage in both cell lines (Figure S1b). TGFβ also induced proteolytic cleavage of CD44 in another non-small-cell lung cancer cell line, H1299, in a time course- and concentration-dependent manner (Figure S1c). The proteolytic cleavage of CD44 was high in U251MG, A549, and H1299 cell lines, compared to other cancer cell lines tested (Figure S1d); these cell lines were therefore selected for further studies.

### 2.2. Stem-Like Cells Show Enhanced Expression of CD44v and HAS2 Genes and Cleavage of CD44

**Figure 2:**
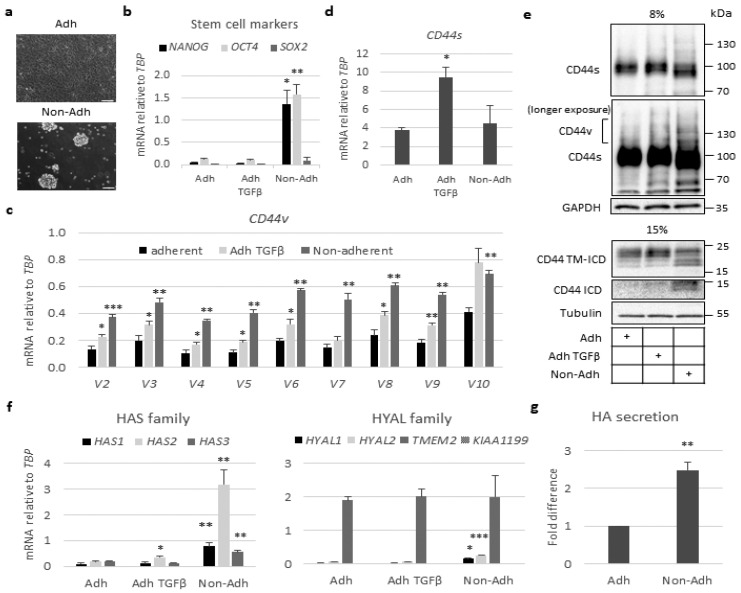
CD44 cleavage increases in stem-like A549 cells grown in spheres. (**a**) Phase contrast microscopy of A549 grown in adherent (Adh) or low-attachment conditions (Non-adh) for 96 h. Zeiss Axiovision was employed to take the micrographs (bars = 100 μm). (**b**–**d**) A549 cells were cultured under adherent conditions or as spheres. Adherent cells were stimulated or not with TGFβ for 24 h. The mRNA levels of transcription factors *NANOG, OCT4,* and *SOX2* (**b**), as well as *CD44v* isoforms (**c**) and *CD44s* (**d**) were determined by real-time qPCR and normalized to *TBP* mRNA levels. (**e**) Lysates from adherent A549 cells, stimulated or not with TGFβ for 24 h, and non-adherent A549 cells were subjected to SDS-PAGE using 8% and 15% polyacrylamide gels, followed by immunoblotting for CD44s and CD44v, CD44 TM-ICD, and CD44 ICD; GAPDH and tubulin were used as loading controls. (**f**) The mRNA levels of hyaluronan synthase (*HAS1*, *HAS2*, *HAS3*) and hyaluronidase (*HYAL1, HYAL2, TMEM2, KIAA1199*) family members were determined by real-time qPCR and normalized to *TBP*. (**g**), Hyaluronan levels in the culture medium of adherent and non-adherent A549 cells were evaluated by an enzyme-linked immunosorbent assay (ELISA)-like assay and normalized against protein amounts; values are expressed as fold difference. Asterisks illustrate significant differences between the different conditions compared to the respective adherent ones: * *p* < 0.05, ** *p* < 0.01, *** *p* < 0.001.

To investigate a possible correlation between CD44 cleavage and stemness in A549 cells, we compared cells cultured in anchorage-dependent monolayer conditions with cells cultured in ultra-low attachment plates, in which tumor cells form spheres enriched in stem-like cancer cells (Figure 2a,b). A549-derived spheres exhibited greater expression of mRNA for the stem cell markers *NANOG* and *OCT4*, compared to cells grown in a monolayer, whereas mRNA for another stem cell marker, *SOX2,* was not increased in the spheres compared to cells grown under adhesive conditions (Figure 2b). The mRNA for the CD44 variant isoforms increased in A549 cell spheres (Figure 2c); however, the transcript for the CD44 standard isoform did not (Figure 2d). Moreover, the expression of CD44v isoforms and the formation of CD44 TM-ICD and CD44 ICD increased in non-adherent A549 cell spheres compared to cells grown in a monolayer (Figure 2e). These findings are consistent with previous reports, which have shown that the cleavage of CD44s and CD44v isoforms are linked to cancer stem-like characteristics [13,33,34,35]. As in A549 cells, the stem cell markers *NANOG* and *OCT4* were also increased in H1299 cells grown under non-adhesive conditions, but the expression levels were about 10-fold lower compared to those in A549 cultures (Figure S2a). Importantly to mention that the expression levels of the two CD44v transcripts (*v3* and *v6*) were lower compared to A549 cells, whereas the mRNA levels for CD44s were similar under adherent and non-adherent conditions (Figure S2b,c); this highlights the correlation of CD44v status with stem cell potential. Quantification of mRNA for *HAS* isoforms in A549 cell spheroids revealed a 30-fold increase in *HAS2* mRNA; the mRNAs for *HAS1* and *HAS3* also increased, but to lesser degrees (Figure 2f). Analysis of the mRNA for hyaluronidase family members revealed that the most abundant one, *TMEM2*, did not increase in the spheroids; however, *HYAL1* and *HYAL2* increased about three-fold (Figure 2f). An about two-fold increase in hyaluronan synthesis in the spheroids, compared to monolayer cell cultures, was observed (Figure 2g). Thus, in stem-like A549 cells grown as spheres, both HAS2-synthesized hyaluronan and CD44v isoforms increased.

Stimulation with TGFβ, which potently induces HAS2-mediated hyaluronan production in several cell types [36,37], enhanced the expressions of mRNAs for *CD44s* and *CD44v*, as well as *HAS2* in A549 cells grown in a monolayer (Figure 2c,d,f).

### 2.3. Involvement of TRAF Family Members in CD44 Cleavage

**Figure 3:**
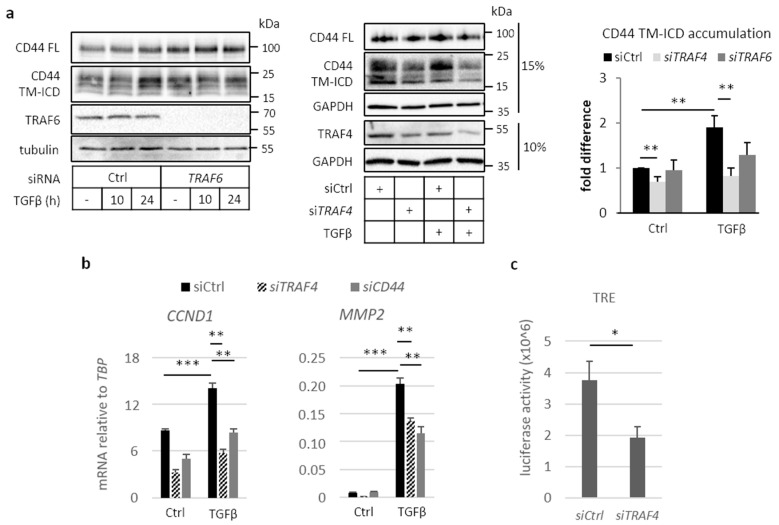
Tumor necrosis factor receptor associated factor (TRAF) family members mediate TGFβ-induced CD44 cleavage. (**a**) A549 cells were transfected with non-targeting siRNA (siCtrl) or siRNAs against *TRAF6* or *TRAF4*, and stimulated or not with TGFβ for up to 24 h, followed by immunoblotting for CD44 FL, TM-ICD, TRAF6, and TRAF4; tubulin and GAPDH served as loading controls. CD44 TM-ICD bands were quantified using the ImageJ software and normalized to either tubulin or GAPDH; values are depicted as fold difference; lysates were run at either 10% or 15% polyacrylamide gels, as depicted. (**b**), A549 cells were transfected with control siRNA or siRNAs targeting *TRAF4* or *CD44*, and subsequently treated or not with TGFβ (5 ng/mL) for 24 h. Relative mRNA levels of CCND1 and MMP2 were quantified by real-time qPCR and normalized to *TBP*. (**c**), A549 cells were co-transfected with scrambled control siRNA or siRNAs against *TRAF4*, a β-gal reporter, and a TPA-responsive element (TRE) luciferase reporter, after which luciferase activity was measured and normalized to β-gal activity. All graph bars are shown as the average ± standard error of the mean (SEM), based on at least three independent experiments, unless mentioned otherwise. Asterisks illustrate significant differences between the conditions indicated with lines; * *p* < 0.05, ** *p* < 0.01, *** *p* < 0.001.

The cleavage of TβRI by ADAM17 and presenilin 1 occurs in a TRAF6-dependent manner [23]. This observation prompted us to investigate whether the cleavage of CD44 is also dependent on TRAF family members. In mouse embryo fibroblasts (MEFs) depleted of *Traf6* (MEF *Traf6^-/-^*), the formation of CD44 TM-ICD was significantly reduced compared to wild-type MEFs (Figure S3a). The ectopic expression of wild-type Traf6 rescued the cleavage products of Cd44 TM-ICD, but the expression of the enzymatically inactive mutant Traf6 C70A did not (Figure S3b,c), suggesting that the cleavage of CD44 is dependent on Traf6-induced ubiquitination. We next investigated the expression levels of mRNA for TRAF family members in A549 and U251MG cells. *TRAF2*, *TRAF4*, *TRAF6*, and *TRAF7* were abundantly expressed in A549 cells, whereas *TRAF4* dominated in U251MG cells (Figure S4a,b); TGFβ stimulation did not affect the expression levels of TRAF family members in either of the cell lines. Silencing of *TRAF6* in A549 cells suppressed the formation of CD44 TM-ICD; however, the silencing of *TRAF4* resulted in an even more prominent reduction of the formation of CD44 TM-ICD (Figure 3a). A decrease in CD44 cleavage was also detected in H1299 cells depleted of TRAF4 (Figure S4c).

The expression of cyclin D1 (*CCND1*) and MMP2 have been shown to be dependent on CD44 [21,38,39]. We found that knockdown of TRAF4 in A549 cells suppressed the TGFβ-induced expression of *CCND1*, as well as *MMP2*, as did knockdown of CD44 (Figure 3b). TRAF4 and CD44 silencing efficiency is depicted in Figure S4d. The translocation of CD44 ICD to the nucleus has been shown to promote activation of the TPA-responsive element (TRE) luciferase reporter [19]; knockdown of TRAF4 reduced the activation of the TRE reporter by 50% (Figure 3c). The results support the notion that TRAF4 is important for CD44 signaling in A549 cells.

### 2.4. TRAF4 Plays an Important Role for CD44 Expression in A549 Cell Spheres

**Figure 4:**
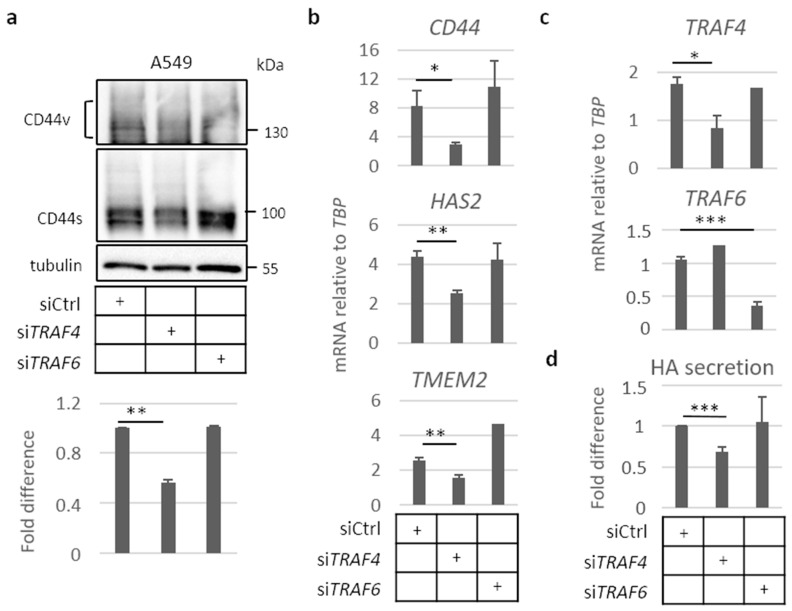
TRAF4, but not TRAF6, is necessary for expression of *CD44* and *HAS2* in A549 cells grown in spheres. A549 cells were transfected with control siRNA or siRNAs targeting *TRAF4* or *TRAF6*, and were thereafter seeded in non-adherent conditions for 96 h. (**a**), Cells were collected, and samples were resolved in 8% SDS-PAGE, followed by immunoblotting for CD44s and v isoforms. CD44 bands were quantified by the software ImageJ and normalized to tubulin levels and expressed as fold difference. (**b**–**c**) mRNA expression of *CD44*, *HAS2*, and *TMEM2* (**b**), as well as *TRAF4* and *TRAF6* (**c**) were determined by real-time qPCR and normalized to *TBP* mRNA. (**d**) Hyaluronan levels in the culture medium of cells in low-attachment conditions was quantified by an ELISA-like assay; the results are given a fold difference after normalization to total protein levels of the collected cells. All graph bars are shown as the average ± SEM based from at least three independent experiments. Asterisks illustrate significant differences compared to the siCtrl condition: * *p* < 0.05, ** *p* < 0.01, *** *p* < 0.001.

We analyzed the importance of TRAF4 and TRAF6 for the expression of CD44s and CD44v, and mRNAs for *CD44*, *HAS2*, and the hyaluronidase *TMEM2* in A549 cell spheres. We found that silencing of TRAF4 by siRNA, but not the silencing of TRAF6, led to about a 40% reduction in the expression of the *CD44s* isoform (Figure 4a). Furthermore, the expression of mRNAs for *CD44*, *HAS2*, and *TMEM2* was suppressed after silencing of TRAF4 (Figure 4b,c), resulting in a 30% decrease in hyaluronan production (Figure 4d). In contrast, knockdown of TRAF6 did not significantly affect the expression of *CD44*, *HAS2*, or *TMEM2,* or the level of secreted hyaluronan (Figure 4b,d, respectively).

### 2.5. TRAF4 Is Required for TGFβ-Induced RAC1 Activation

**Figure 5:**
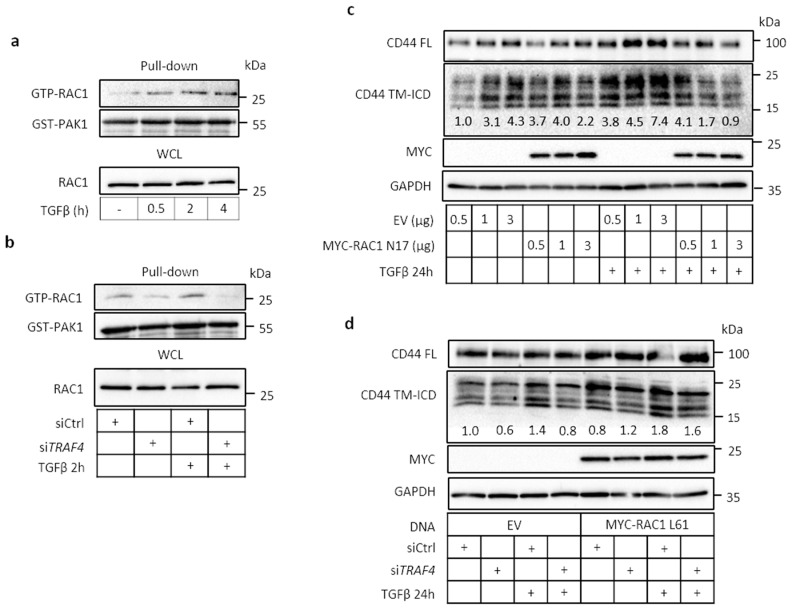
TRAF4 is required for TGFβ-induced RAC1 activity and for CD44 cleavage. (**a**) A549 cells were treated with TGFβ (5 ng/mL) for the indicated time periods, and RAC1 activity was assessed after pull-down with GST-PAK1, followed by immunoblotting for RAC1 and GST-PAK1; in addition, whole cell lysate was immunoblotted for RAC1. (**b**), A549 cells were transfected with control siRNA or TRAF4 siRNA, and treated with TGFβ (5 ng/mL) for 2 h. RAC1 activity was determined as in panel a. (**c,d**), A549 cells were transfected with either empty vector, different amounts of RAC1 dominant-negative mutant (MYC-RAC1 N17) (**c**), or MYC-tagged RAC1 constitutively active mutant (MYC-RAC1 L61) (**d**), followed by treatment with or without TGFβ (5 ng/mL) for 24 h. Cell lysates were subjected to immunoblotting for MYC, FL CD44 and CD44 TM-ICD. GAPDH was used as loading control. Cells transfected with non-targeting siRNA or siRNAs against *TRAF4* were also analyzed (**d**). CD44 TM-ICD bands were quantified using the ImageJ software and normalized to GAPDH; values are shown as fold-difference.

To further address the mechanisms regulating signaling via CD44 and TRAF4, we investigated the involvement of the small GTPase RAC1, which has been demonstrated to promote the cleavage of CD44 in TPA- [12] or EGF-stimulated [40] U251MG cells. TGFβ stimulation of A549 cells activated RAC1, first observed after 30 min and sustained over several hours, as demonstrated by the pull-down of GTP-RAC1 by glutathione S-transfarase-tagged p21-activated kinase (GST-PAK1) (Figure 5a). Silencing of TRAF4 by siRNA inhibited the activation of RAC1 (Figure 5b). Transfection of a dominant-negative RAC1 mutant (MYC-RAC1 T17N) suppressed the TGFβ-induced formation of CD44 TM-ICD (Figure 5c), as did knockdown of TRAF4 (Figure 5d). In contrast, transfection of a constitutively active RAC1 mutant (MYC-RAC1 Q61L) rescued the formation of CD44 TM-ICD, in the absence or presence of TGFβ (Figure 5d). A similar dependence on TRAF4 for RAC1 activation was also observed in H1299 cells (Figure S5). Moreover, TGFβ-mediated cleavage of CD44 was suppressed by the dominant-negative mutant MYC-RAC1 N17 (Figure S5c).

### 2.6. Migration of A549 Cells Is Dependent on CD44, TRAF4, and RAC1

**Figure 6:**
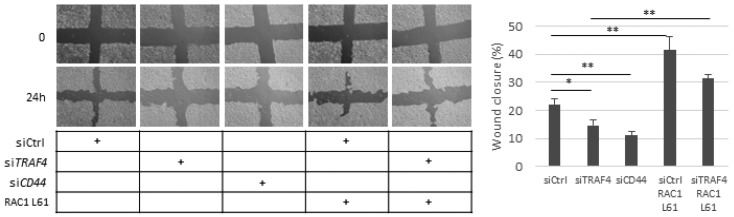
Knockdown of TRAF4 diminishes cell motility, which is rescued by constitutively active RAC1. A549 cells were transfected with control siRNA or siRNAs targeting *TRAF4* or *CD44* and co-transfected with either empty vector or the constitutively active RAC1 L61 mutant. Cell migration was evaluated using a cell culture wound healing assay. Pictures of the cell cultures were taken at 0 h and at 24 h. The results are presented as a percentage of wound closure and illustrate the average values ± SEM out of three independent experiments. Asterisks illustrate significant differences between the conditions indicated with lines: * *p* < 0.05, ** *p* < 0.01.

In order to determine if CD44-mediated migration of A549 cells requires TRAF4 and RAC1, we employed a cell culture scratch assay. The silencing of either CD44 or TRAF4 decreased the migration of A549 cells significantly; ectopic expression of the constitutively active RAC1 L61 rescued the migration of TRAF4-depleted cells and enhanced the migration of control cells (Figure 6). This observation suggests that the TRAF4–RAC1 axis might be important for CD44-dependent A549 cell motility. The migration of H1299 cells was significantly reduced by knockdown of CD44; a reduced migration, albeit not significant, was also observed in TRAF4-depleted cells, probably due to insufficient silencing efficiency (Figure S6).

## 3. Discussion

**Figure 7:**
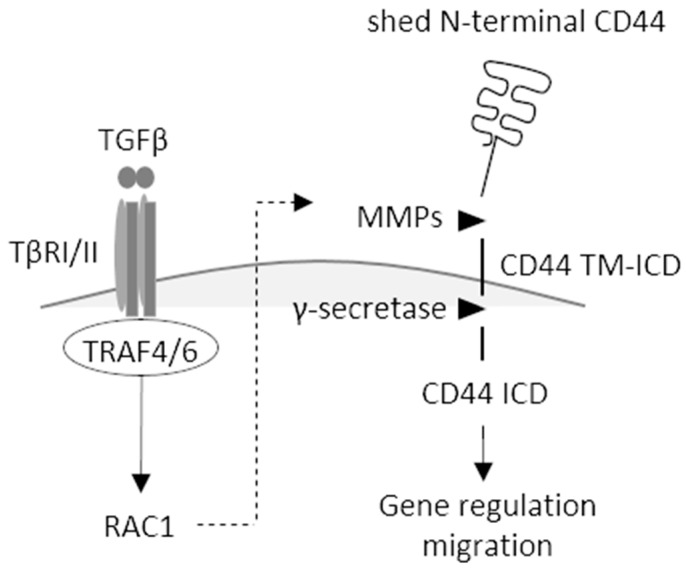
Schematic illustration of TGFβ-induced activation of TRAF4/6 and RAC1, leading to CD44 cleavage, enhanced migration, and altered gene regulation. Binding of TGFβ to its type II receptor (TβII) recruits and activates the type I receptor (TβRI), activating TRAF4/6 and RAC1, leading to the activation of MMPs, resulting in the formation of CD44 TM-ICD; after further processing by γ-secretase, CD44 ICD is translocated to the nucleus, where it regulates transcription and promotes cell migration.

We show in the present communication that TRAF4 is needed for the activation of RAC1 and cleavage of CD44, resulting in the formation of CD44 TM-ICD and CD44 ICD and promoting the migration of A549 cells. It is possible that TRAF4 activation and other signaling pathways lead to RAC1-mediated activation of MMPs, enhancing CD44 cleavage (Figure 7) [14]. TRAF4 is also required for the increased expression of CD44 variant isoforms and HAS2 in stem-like cells, grown as spheres, leading to increased synthesis of hyaluronan.

TRAF6 has been shown to be essential for TGFβ-stimulated, two-step cleavage of TβRI, resulting in the release of TβRI-ICD [23]. We found that TRAF6 is important also for the cleavage of CD44 and release of its intracellular domain in MEFs, as well as in A549 cells; however, in A549 cells, TRAF4 was found to be more important (Figure 3). The expression of full-length (FL) CD44 in MEF Traf6^-/-^ was lower than in MEF wild-type (Wt) (Figure S3). According to previous reports, upon ICD translocation to the nucleus, *CD44* is among the positively regulated genes. Due to stable depletion of Traf6, CD44 cleavage is constantly impaired, therefore disrupting the positive feedback loop replenishing Cd44 FL. Thus, it is possible that this would lead to a significant downregulation of Cd44 in Traf6^-/-^ cells. Since TRAF4 and TRAF6 are structurally related to each other, it is possible that they have partly redundant functions, and that their relative expression levels determine which of the two isoforms is the most important for the cleavage of CD44 in different cell types. TRAF4 has been found to promote TGFβ signaling via Smad and non-Smad pathways [28], and to promote the migration of hepatocellular carcinoma [41], as well as endothelial and epithelial cells [42,43]. Moreover, *TRAF4* has been found to be amplified during breast cancer progression [44]. Thus, TRAF4 has pro-tumorigenic effects in advanced cancer.

Cleavage of CD44 has been observed in several types of tumors, and increases in response to stimulation with fragmented hyaluronan, activation of protein kinase C (PKC), and activation of RAS [45,46,47], and CD44 ICD has been shown to promote epithelial–mesenchymal transition and the stemness of tumor cells [34]. Furthermore, CD44 ICD drives the proliferation of thyroid cancer by increasing cyclin D1 expression and the activity of CREB [38]. Mechanistically, CD44 ICD has been shown to bind to a consensus sequence, CCTGCG, found for example in the promoter regions of the *MMP9* and *HIF1a* genes, and to interact with the co-activators CBP/p300 [19,38,48]. It has also been reported that CD44 ICD confers hypoxia-inducible factor-2α (HIF2α) stabilization, leading to an enhanced hypoxia-related gene signature [49]. Moreover, a recent study highlighted the importance of CD44 and its cleavage products in driving PD-L1 expression in A549 cells [50]. Taken together, these observations support the notion that CD44 ICD has a tumor promoting role. We observed that both TRAF4 and CD44 are needed for TGFβ stimulated induction of *CCND1* (Figure 3). Our findings support the notion that TRAF4/6 promote pro-tumorigenic effects of CD44.

The expression of CD44v isoforms is linked to tumor growth, metastasis, and stemness [35], and importantly, CD44v isoforms exhibit increased proteolytic cleavage compared with CD44s in tumors [47]. CD44v6 is a biomarker associated with colon cancer stemness and tumor progression [51,52], and hyaluronan-engaged CD44v3 induces the expression of stem cell markers, NANOG, SOX2, and OCT4, promoting tumor progression and chemoresistance of head and neck squamous cell carcinomas [18,53]. Consistent with these observations, we found that CD44v isoforms are overexpressed in A549 cells grown as spheres and enriched for stemness markers, such as *NANOG* and *OCT4* (Figure 2). We also found that A549 cells grown in spheres overexpress HAS2 and secrete more hyaluronan than cells grown under adhesive conditions (Figure 2f,g). Interestingly, hyaluronan synthesis was found to be increased in spheroids compared to adherent cultures of malignant mesothelioma [54]. These observations are consistent with the possibility that hyaluronan contributes to the stem cell niche [55].

The findings reported in this communication support the notion that TRAF4 mediates CD44 cleavage through the activation of RAC1, which is required for the migration of A549 cells. We also demonstrate a TRAF4-dependent association between tumor cell stemness, CD44v expression, and increased synthesis of hyaluronan. Our results suggest that the inhibition of CD44 signaling via TRAF4 and RAC1 may be beneficial in tumor treatment.

## 4. Materials and Methods

### 4.1. Cell Culture, Transfections, Plasmids, and Reagents

Human lung adenocarcinoma A549 cells (kindly provided by A. Moustakas, Uppsala University, purchased from American Type Culture Collection (ATCC)), non-small-cell lung carcinoma H1299 cells [56], glioblastoma U251MG cells [57], and prostate cancer PC3U cells [23] were grown in RPMI-1640 (Sigma-Aldrich Sweden AB, Stockholm, Sweden), whereas wild-type (Wt) or Traf6-deficient (*Traf6*^-/-^) mouse embryonic fibroblasts [23], human breast cancer Hs578 cells [56], and a bone-metastasizing clone of human breast cancer MDA MB 231 cells [58] were cultured in Dulbecco’s Modified Eagle Medium (DMEM) (Sigma-Aldrich Sweden AB, Stockholm, Sweden), all in the presence of 10% fetal bovine serum (FBS) (Biowest, Biotech-IgG AB, Lund, Sweden), penicillin and streptomycin (100 X/mL), and 5 mM L-glutamine (Sigma-Aldrich Sweden AB, Stockholm, Sweden). Human colon cancer HCT-116 and CACO-2 cells were grown in McCoy′s 5a medium (Sigma-Aldrich Sweden AB, Stockholm, Sweden) and in Minimum Essential Medium Eagle (Sigma-Aldrich Sweden AB, Stockholm, Sweden), respectively (both cell lines were kindly provided by Dr I. Ferby, Uppsala University, being purchased from ATCC). Ras-transformed premalignant MCF10AneoT (MII) cells [59] were cultured in DMEM/F12 (Gibco, Life Technologies Europe BV, Stockholm, Sweden). Cells were maintained at 5% CO_2_ in a humidified atmosphere at 37 °C.

For culturing in non-adherent conditions, A549 or H1299 cells were grown for 96 h in ultra-low-attachment six-well plates (Corning, Sigma-Aldrich Sweden AB, Stockholm, Sweden) in DMEM/F12 medium, supplemented with B27-containing vitamin A (Invitrogen, Life Technologies Europe BV, Stockholm, Sweden), also including basic fibroblast growth factor (20 ng/mL), EGF (20 ng/mL), insulin (4 μg/mL), and TGFβ (10 ng/mL) (PeproTech EC Ltd. Nordic, Stockholm, Sweden). Treatments with tumor necrosis factor alpha, hepatocyte growth factor, stem cell factor, and platelet-derived growth factor-BB (all from PeproTech EC Ltd. Nordic, Stockholm, Sweden; or Biosource Inc., Dacula, GA, United States) were at 10 ng/mL. The siRNAs (Non-targeting control, *TRAF4*, *TRAF6*, and *CD44* (all ON-TARGET plus) were purchased from Dharmacon, Thermo Fischer Scientific (Gothenburg, Sweden), and transiently transfected on two consecutive days, 30 nM each time. Silentfect (Biorad Laboratories AB, Sundbyberg, Sweden) was used as a liposome reagent, according to the manufacturer’s instructions. For cDNA transfections, Lipofectamine 3000 reagent (Thermo Fischer Scientific, Gothenburg, Sweden) was employed, following the manufacturer’s protocol. FLAG-tagged TRAF6 Wt and TRAF6 C70A plasmids were kind gifts from Dr M. Landström, Umeå University. MYC-tagged RAC1 T17N, which is a nucleotide binding-defective mutant, hence acting in a dominant-negative (DN) manner, and RAC1 Q61L, which is a defective GTPase and thus acts in a constitutively active (CA) manner [60,61], were kind gifts from Dr P. Aspenström, Uppsala University. The proteasomal inhibitor MG132 was purchased from Calbiochem, Merck, Germany, and the lysosomal inhibitor chloroquine, the γ-secretase inhibitor DAPT, and 12-O-tetradecanoylphorbol-13-acetate (TPA) from Sigma-Aldrich Sweden AB, Stockholm, Sweden. High-molecular-weight hyaluronan (HMW HA; 1000 kDa) was kindly provided by Dr. Ove Wik (Q-Med, Uppsala, Sweden), as was 200 kDA of fragmented hyaluronan by Dr A. Passi (Insurbia University, Varese, Italy).

### 4.2. Immunoblotting

Cells were lysed in buffer containing 50 mM Tris-HCl, pH 8.0, 150 mM NaCl, 1% NP-40, 0.1% sodium dodecyl sulfate (SDS), and 0.5% sodium deoxycholate, supplemented with HALT protease and a phosphatase inhibitor cocktail (Thermo Fischer Scientific, Gothenburg, Sweden); protein concentration was measured by utilizing a Bicinchoninic acid (BCA) assay (Thermo Fischer Scientific, Gothenburg, Sweden). Samples with equal protein content were subjected to SDS–polyacrylamide gel electrophoresis (SDS-PAGE), followed by wet transfer to nitrocellulose membrane (Amersham, GE Healthcare, Uppsala, Sweden) and blocking in 5% non-fat milk in Tris buffered saline (TBS), supplemented with 1% Tween 20. Subsequently, the membranes were incubated at 4 °C overnight with antibodies against CD44 (ab157107), lamin (ab16048) (Abcam, Cambridge, United Kingdom), α-tubulin (TU-02), MYC (SC-40), RAC1 (C-11), GST (B-14) (Santa Cruz Biotechnology Inc., Santa Cruz, CA, United States), GAPDH (D16H11) and phospho-SMAD3 (9520) (Cell Signaling Technology, Leiden, The Netherlands), TRAF4 (BD Biosciences, Stockholm, Sweden), or TRAF6 (Invitrogen, Life Technologies Europe BV, Stockholm, Sweden)), followed by incubation with horseradish peroxidase-conjugated secondary antibodies (1:10,000; Invitrogen, Life Technologies Europe BV, Stockholm, Sweden) for 1 h at room temperature, and development by chemiluminescence (Millipore, MA, United States). Band intensity quantification was performed by using the ImageJ software.

### 4.3. Nuclear–Cytoplasmic Fractionation

For the isolation of cytoplasmic and nuclear fractions from A549 cell cultures, NuCLEAR Extraction Kit (Sigma-Aldrich Sweden AB, Stockholm, Sweden) was employed following the manufacturer’s instructions. Briefly, cells from one 10-cm petri dish per condition were centrifuged, and cell pellets were incubated with 300 μL hypotonic lysis buffer (cytoplasmic fraction). Nuclei were resuspended in 100 μL of nuclear extraction buffer. For immunoblotting, 30 μL of the cytoplasmic fraction and 10 μL of the nuclear were subjected to electroforesis in either 10% or 15% polyacrylamide gels. Lamin B1 and tubulin were used as markers for nuclear and cytoplasmic compartments, respectively.

### 4.4. RAC1 Activity Assay

After the indicated treatments, A549 cells were washed once in ice-cold PBS and lysed in 50 mM Tris-HCl, pH 7.5, 1% Triton X-100, 0.5% deoxycholate, 500 mM NaCl, and 10 mM MgCl_2_ supplemented with protease and phosphatase inhibitors. Cell lysates were centrifuged at 13,000 rpm for 5 min; thereafter, supernatants were incubated with GST-fused PAK-1 (kindly provided by Dr P. Aspenström, Uppsala University) isolated from bacteria after pulling down with glutathione-fused agarose beads for 10 min at 4 °C, end over end. Afterwards, the beads were washed three times with wash buffer (50 mM Tris-HCl, pH 7.5, 1% Triton X-100, 150 mM NaCl, 10 mM MgCl_2_) and eluents were retrieved in 2x Laemmli sample buffer. Pulled down GTP-bound RAC1 was detected by immunoblotting analysis.

### 4.5. RNA Extraction and Real-Time qPCR

RNA was extracted from cells by using the RNeasy kit (QIAGEN AB, Sollentuna, Sweden) according to the manufacturer’s instructions. An iScript DNA synthesis kit (Biorad, Biorad Laboratories AB, Sundbyberg, Sweden) was used to reverse-transcribe 1 μg of total RNA to cDNA. KAPA Sybr Fast (PCR biosystems, Techtum Lab AB, Umeå, Sweden) was employed to perform real-time qPCR in triplicate (95 °C, 2 min; 40 × (95 °C, 10 s; 60 °C, 30 s)). Primers used for gene detection are summarized in Table S1. Gene expression was normalized to the housekeeping gene *TATA-Box Binding Protein* (*TBP)*.

### 4.6. Luciferase Assay

A549 cells were co-transfected with siRNA targeting *TRAF4* or control siRNA, together with the 5xTRE reporter plasmid and pCMV-β-gal (a β-galactosidase expression plasmid), kind gifts from Drs H. van Dam (Leiden University, Holland) and A. Moustakas (Uppsala University, Sweden), respectively. The β-galactosidase assay was performed by mixing the cell lysate with 100 mM sodium phosphate pH 7.3, 1 mM MgCl_2_, 50 mM β-mercaptoethanol, and 0.67 mg/mL of 2-nitrophenyl β-D-galactopyranoside, followed by monitoring of the absorbance at 420 nm. Luciferase reporter assays were performed with the firefly luciferase assay kit from Biotium (Fremont, CA, United States (BTIU30003-2)), according to the protocol of the manufacturer.

### 4.7. Phase Contrast Microscopy

Cells were subjected to phase contrast microscopy by employing a Zeiss Axiovision 40 microscope (Carl Zeiss) at the indicated time points and conditions. Bars at the respective micrographs represent 100 μm.

### 4.8. In Vitro Wound Healing Assay

A549 or H1299 cells were treated with siRNAs as indicated, and then grown to confluency. One vertical and three parallel horizontal wounds were then introduced by using a 200 μL pipette tip. Fresh medium containing 5% FBS was added and wound closure was monitored with a Zeiss Axiovert 40 phase-contrast microscope after 24 h, and quantified by the ImageJ software.

### 4.9. Hyaluronan Assay

Cell culture media from A549 and U251MG cultures, conditioned for 24 h, were collected after the indicated treatments, and hyaluronan concentration was measured and normalized to 1 μg of total protein extracted from the cells, as previously described [37].

### 4.10. Statistical Analysis

Graphs showing the mean ± SEM and are based on at least three independent experiments, unless stated otherwise. Two-paired Student’s *t*-test was used to calculate significance; three significance levels are indicated, i.e., * *p* < 0.05, ** *p* < 0.01, and *** *p* < 0.001.

## 5. Conclusions

The cleavage of the hyaluronan receptor CD44 is mediated by TRAF4 via the activation of RAC1, promoting migration of A549 lung cancer cells. The expression of CD44 variant isoforms, the cleavage of CD44, and the production of hyaluronan increases in stem-like tumor cells grown in spheres.

## Data Availability

No data more than what is shown in this study were created or analyzed. Data sharing is not applicable to this article.

## Supplementary Materials

The following are available online at https://www.mdpi.com/2072-6694/13/5/1021/s1, Figure S1: Screening for CD44 cleavage in different cell lines and in response to various external stimuli; Figure S2: *NANOG*, *OCT4*, and *CD44v3* and *v6* increase in stem-like H1299 cells grown in low-attachment conditions; Figure S3: The catalytic activity of TRAF6 is indispensable for TGFβ-induced CD44 cleavage in mouse embryo fibroblasts (MEFs); Figure S4: Importance of TRAF4 for the cleavage of CD44 in H1299 cells and expression of TRAF family members in lung cancer and glioma cells; Figure S5: TRAF4 is required for TGFβ-induced RAC1 activity and subsequent CD44 cleavage in H1299; Figure S6: Knockdown of CD44 decreases cell motility of H1299, Table S1: Primers used in this study.
