# Supplementary material for: TRAF4/6 Is Needed for CD44 Cleavage and Migration via RAC1 Activation
Source: Cancers (Basel). 2021 Mar 1;13(5):1021. doi: 10.3390/cancers13051021 (PMC7957764; doi:10.3390/cancers13051021)
Supplement: Supplementary file 1 [file cancers-13-01021-s001.zip › supp+WB/cancers-1048357 proofed supp.docx]

**Supplementary files R1 cancers-1048357 Kolliopoulos et al.**


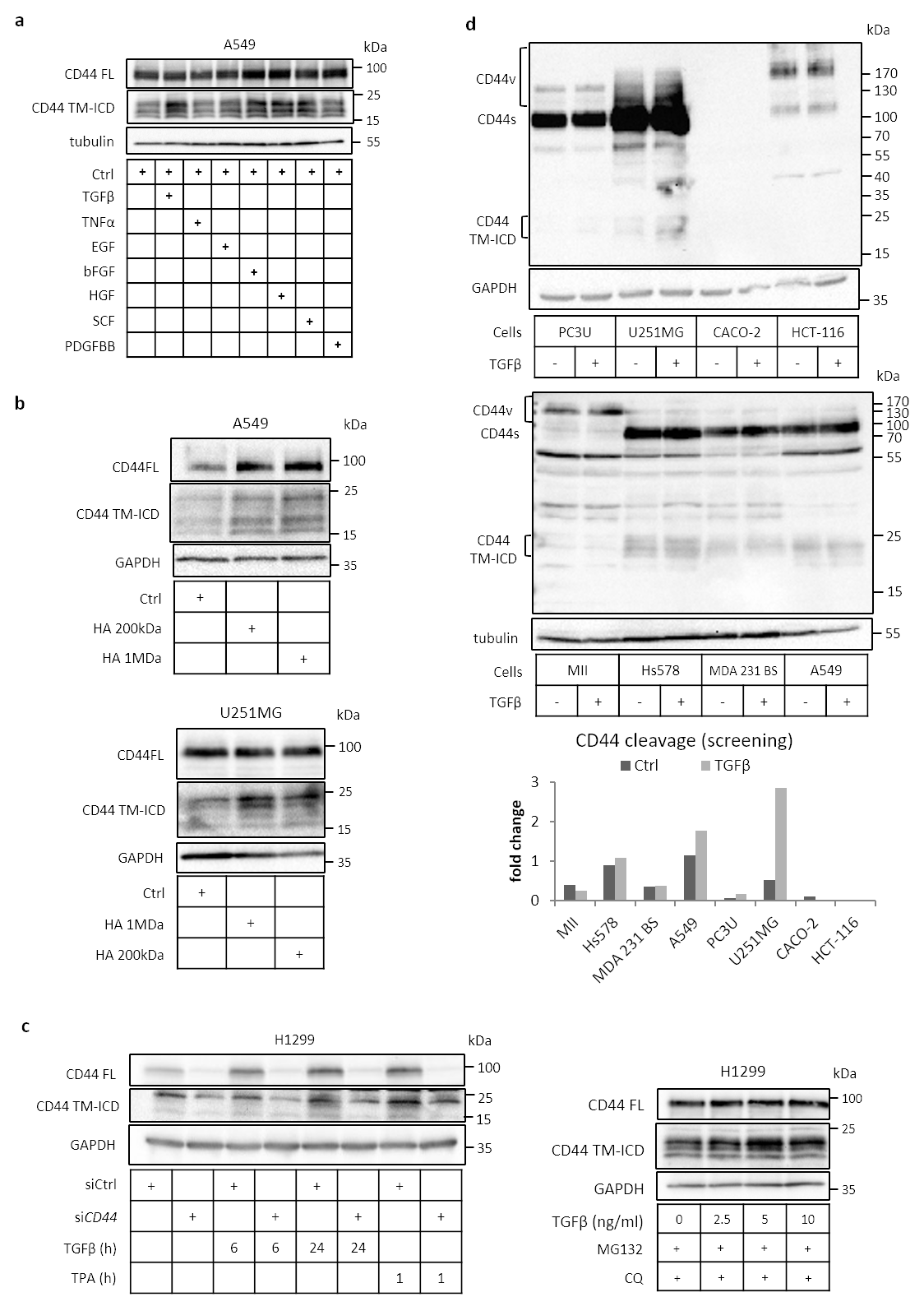


**Figure S1. Screening for CD44 cleavage in different cell lines and in response to various external stimuli**. (**a**), A549 cells were stimulated for 24 h with various growth factors, and thereafter subjected to immunoblotting for FL and TM-ICD CD44; tubulin was used as loading control. (**b**), A549 and U251MG cells were treated with hyaluronan (HA) of different sizes, and thereafter subjected to immunoblotting for FL and TM-ICD CD44. (**c**) Immunoblot analysis of FL and TM-ICD CD44 in H1299 cells. H1299 cells were transfected with control siRNA or siRNA targeting *CD44* and were treated with or without TGFβ (5 ng/ml), MG132 (40 μM) and CQ (40 μM) for 24 h, or TPA (80 nM) for 1 h, followed by immunoblotting for CD44FL, TM-ICD, and GAPDH. (**d**), Prostate cancer cells PC3U, glioblastoma cells U251MG, colon adenocarcinoma CACO-2, colon cancer HCT-116, breast cancer cells MCF10AneoT MII, triple negative breast cancer cells Hs578 and MDA 231 bone-metastasizing clone (MDA 231 BS) and lung adenocarcinoma A549 were treated or not with TGFβ (5 ng/ml) for 24 h after being starved overnight, and were subjected to SDS-PAGE and subsequent immunoblotting analysis to assess levels of CD44s, CD44v isoforms, and their cleavage products (CD44 TM-ICD). GAPDH or tubulin immunoblots served as loading controls. Band intensities were quantified by the software ImageJ and results were normalized to loading control.

**
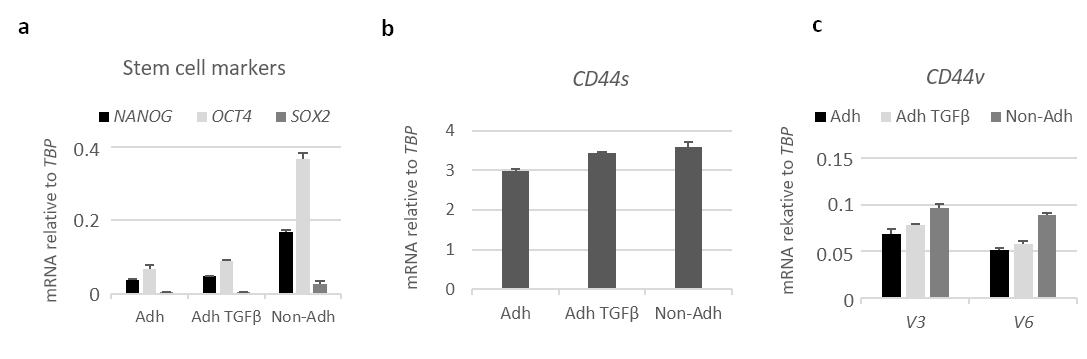
 Figure S2. *NANOG*, *OCT4* and *CD44v3 and v6* increases in stem-like H1299 cells grown in low-attachment conditions.** (**a-c**) H1299 cells were seeded under adherent conditions or non-adherent in ultra-low attachment plates. Adherent cells were treated or not with TGFβ for 24 h. mRNA levels of transcription factors *NANOG, OCT4, SOX2* (a), *CD44s* (b) and *CD44v* isoforms *V3* and *V6* (c) were determined by real-time qPCR and normalized to *TBP* mRNA levels.


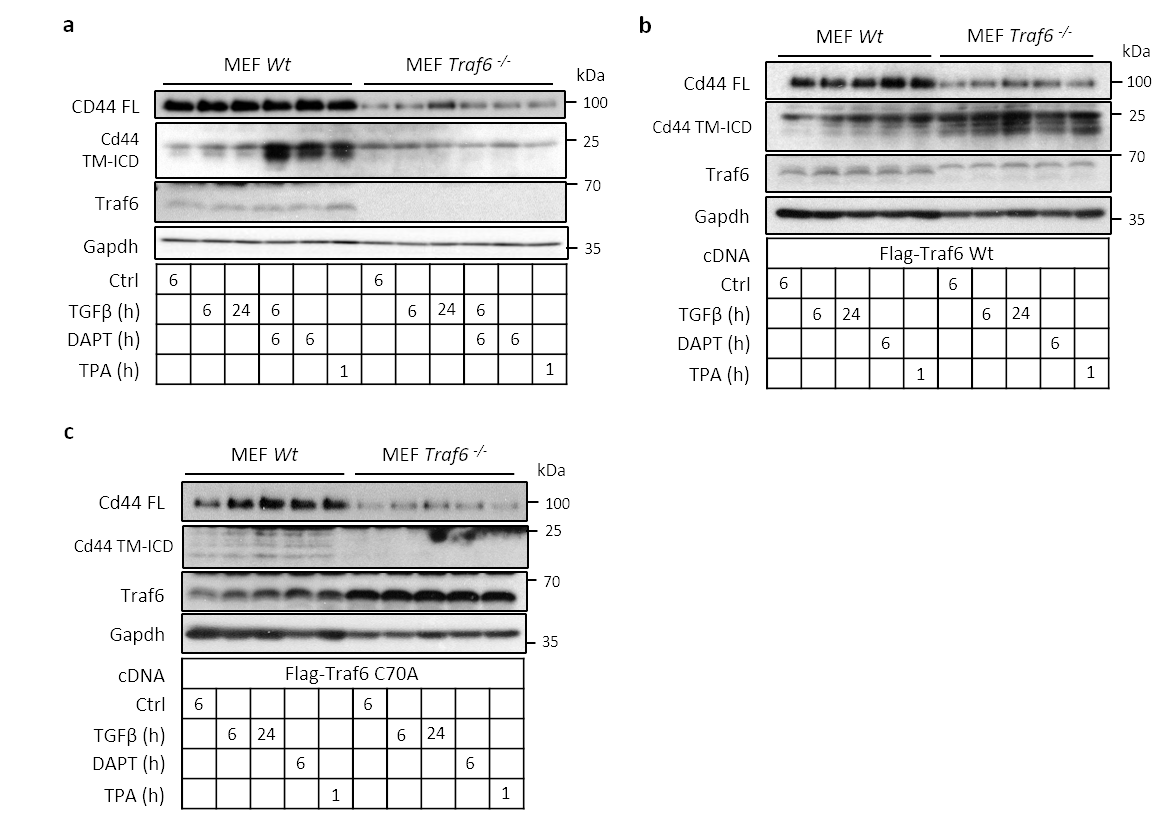


**Figure S3. The catalytic activity of Traf6 is indispensable for TGFβ-induced CD44 cleavage in mouse embryo fibroblasts (MEF).** (**a**), Immunoblotting for FL and TM-ICD Cd44, and Traf6, in wild-type (Wt) and Traf6-depleted (*Traf6* ^-/-^) MEF cells, treated or not with TGFβ (5 ng/ml), DAPT (40 μM) and TPA (80 nM) for the indicated time periods. (**b**), Wt MEFs and MEFs depleted of Traf6 (*Traf6* ^-/-^) were transfected with Wt Traf6 cDNA. Subsequently cells were treated or not with TGFβ (5 ng/ml) for the indicated time periods or with DAPT (30 μM) for 6 h or TPA (80 nM) for 1 h, and subjected to immunoblotting for FL and TM-ICD Cd44, Traf6 and Gapdh. (**c**), Wt and *Traf6* ^-/-^ MEFs were transfected with catalytically inactive mutant Flag-Traf6 C70A cDNA, and were subjected to analyses as in panel (b). Immunoblots were re-probed for Gapdh which was used as a loading control.


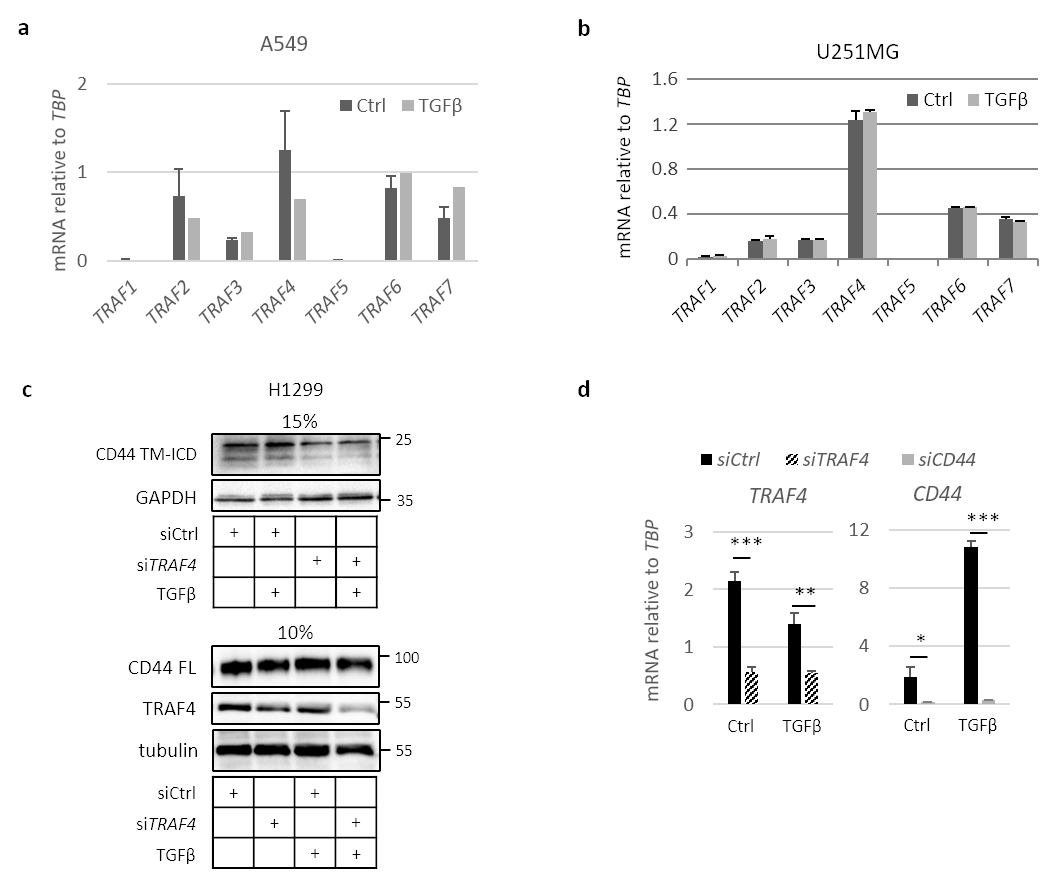


**Figure S4.** Importance of TRAF4 for the cleavage of CD44 in H1299 cells and expression of TRAF family members in lung cancer and glioma cells. (a, b), mRNA levels of TRAF family members in A549 cells and U251MG cells (b) stimulated or not with TGFβ for 24 h, were determined by real-time qPCR and normalized to *TBP*. (c), Lysates from H1299 cells, transfected or not with *TRAF4* siRNA, and stimulated or not with TGFβ for 24 h, were subjected to immunoblotting for CD44 FL and TM-ICD; GAPDH and tubulin served as loading controls. (d) depicts the silencing efficiency of TRAF4 and CD44 in A549 cells untreated or treated with TGFβ. *p<0.05, ***p<0.001.


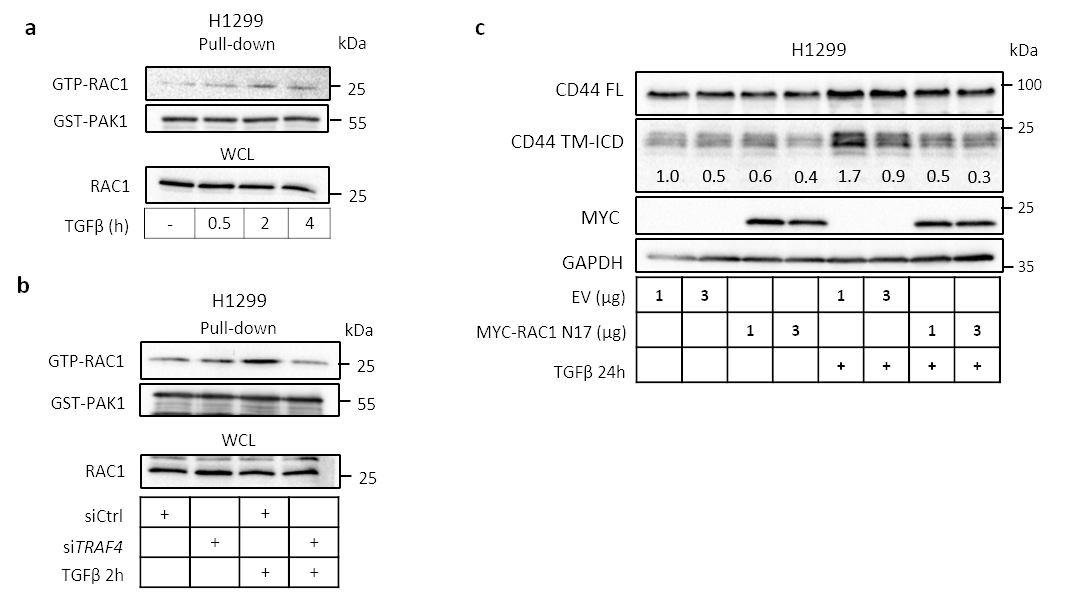


**Figure S5. TRAF4 is required for TGFβ-induced RAC1 activity and subsequent CD44 cleavage in H1299.** (**a**), H1299 cells were stimulated with TGFβ (5 ng/ml) for the indicated time periods and RAC1 activity was determined after pull-down with GST-PAK1, followed by immunoblotting for RAC1 and GST-PAK1; Whole cell lysate was immunoblotted for RAC1. (**b**), H1299 cells were transfected with non-targeting siRNA (siCtrl) or *TRAF4* siRNA, and treated with TGFβ (5 ng/ml) for 2 h. RAC1 activity was determined as in panel a. (**c**), H1299 cells were transfected with either empty vector or various amounts of a RAC1 dominant negative mutant (MYC-RAC1 N17) in the presence or absence of TGFβ for 24 h. Cell lysates were subjected to immunoblotting for FL CD44 and CD44 TM-ICD. GAPDH was used as loading control. CD44 TM-ICD bands were determined using the ImageJ software and normalized to GAPDH; values are shown as fold-difference.


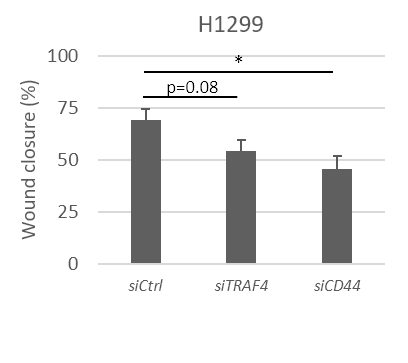


**Figure 6. Knock-down of CD44 decrease cell motility of H1299 cells.** Wound healing assay was performed on cells transfected with control siRNA or siRNAs targeting *TRAF4* or *CD44*. The results are shown as percentage of wound closure and demonstrate average values ± SEM out of at least three independent experiments. Asterisks illustrate significant differences between the conditions indicated with lines; *p<0.05.

**Table 1. Primers used in this study.**

| *Gene* | 5’ Primer sequence | 3’ Primer sequence |
| --- | --- | --- |
| *TBP* | TGGCGTGTGAAGATAACCCAA | TCTTGGCAAACCAGAAACCCT |
| *CD44S* | ATAATAAAGGAGCAGCACTTCAGGA | ATAATTGTGTCTTGGTCTCTGGTAGC |
| *CD44V2* | ATAATCAGCAACTGAGACAGCAACCAA | ATTATAACCAATCCCAGGTTTCTTGCC |
| *CD44V3* | ATAATGGCTGGGAGCCAAATGAAGAAA | ATAATCATCATCATCAATGCCTGATCCAGA |
| *CD44V4* | ATAATCAGTGGAACCCAAGCATTCAA | ATAATCCTTGTGGTTGTCTGAAGTAGCAC |
| *CD44V5* | ATAATGAAACTGGAACCCAGAAGCACA | ATAATTGATGCTCATGGTGAATGAGGG |
| *CD44V6* | ATAATCAGAAGGAACAGTGGTTTGGCA | ATAATGTCTTCTTTGGGTGTTTGGCGA |
| *CD44V7* | ATAATTGCAAGGAAGGACAACACCAAG | ATAATGGGTGTGAGATTGGGTTGAAGA |
| *CD44V8* | ATAATACGCTTCAGCCTACTGCAAA | ATAATAAGAGGTCCTGTCCTGTCCAAA |
| *CD44V9* | ATAATGAGCTTCTCTACATCACATGAAGGC | ATAATGTCAGAGTAGAAGTTGTTGGATGGTC |
| *CD44V10* | ATAATACCTCTCATTACCCACACACGA | ATAATTAGCTGAGGTCACTGGGATGAA |
| *CD44* total | ATAATTGCCGCTTTGCAGGTGTATT | ATAATGGCAAGGTGCTATTGAAAGCCT |
| *NANOG* | GCTGGTTGCCTCATGTTATTATGC | CCATGGAGGAAGGAAGAGGAGAGA |
| *POUF5F1* | AGCAAAACCCGGAGGAGT | CCACATCGGCCTGTGTATATC |
| *SOX2* | GCCTGGGCGCCGAGTGGA | GGGCGAGCCGTTCATGTAGGTCTG |
| *MMP2* | GTGCTGAAGGACACACTAAAGAAGA | TTGCCATCCTTCTCAAAGTTGTAGG |
| *CCND1* | AGCTCCTGTGCTGCGAAGTGGAAA | AGTGTTCAATGAAATCGTGCGGGGT |
| *HAS1* | GGAATAACCTCTTGCAGCAGTTTC | GCCGGTCATCCCCAAAAG |
| *HAS2* | TCGCAACACGTAACGCAAT | ACTTCTCTTTTTCCACCCCATTT |
| *HAS3* | AACAAGTACGACTCATGGATTTCCT | GCCCGCTCCACGTTGA |
| *HYAL1* | GATGTCAGTGTCTTCGATGTGGTA | GGGAGCTATAGAAAATTGTCATGTCA |
| *HYAL2* | CTAATGAGGGTTTTGTGAACCAGAATAT | GCAGAATCGAAGCGTGGATAC |
| *TMEM2* | GGAATAGGACTGACCTTTGCCAG | TTCTGACCACCCTGAAAGCCGT |
| *KIAA1199* | ACCGAGCACATTCCAACTACCG | GGCAGAGATGATTGAGAGGAACG |
| *TRAF1* | CTTGGAGCAGAGGGTGGT | GCCTGGTGACATTGGTGAT |
| *TRAF2* | AACATTGTCTGCGTCCTGAACC | AGCCATCGCCAGGTCCTTG |
| *TRAF3* | CGAGCGGCGACGGAC | CGCCAGGAGAGTCCATCTTTT |
| *TRAF4* | GCTGCATCCACAGTGAGGAGGG | CTCAGAGGTGGCATGCTGGGCC |
| *TRAF5* | CGCACGTGAGGGAAATCAGA | CCGCTCCACAAACTGGTACT |
| *TRAF6* | CTGCCCTACAGCCCCAATTC | CAAGGCGACCCTCTAACTGG |
| *TRAF7* | GTTCATCGGGAACCAGGACAC | GATCTTCTCCGAGAGCTTTCCC |
