# Supplementary material for: TRAF4/6 Is Needed for CD44 Cleavage and Migration via RAC1 Activation
Source: Cancers (Basel). 2021 Mar 1;13(5):1021. doi: 10.3390/cancers13051021 (PMC7957764; doi:10.3390/cancers13051021)
Supplement: Supplementary file 1 [file cancers-13-01021-s001.zip › supp+WB/cancers-1048357 WB.pdf]

Figure 1

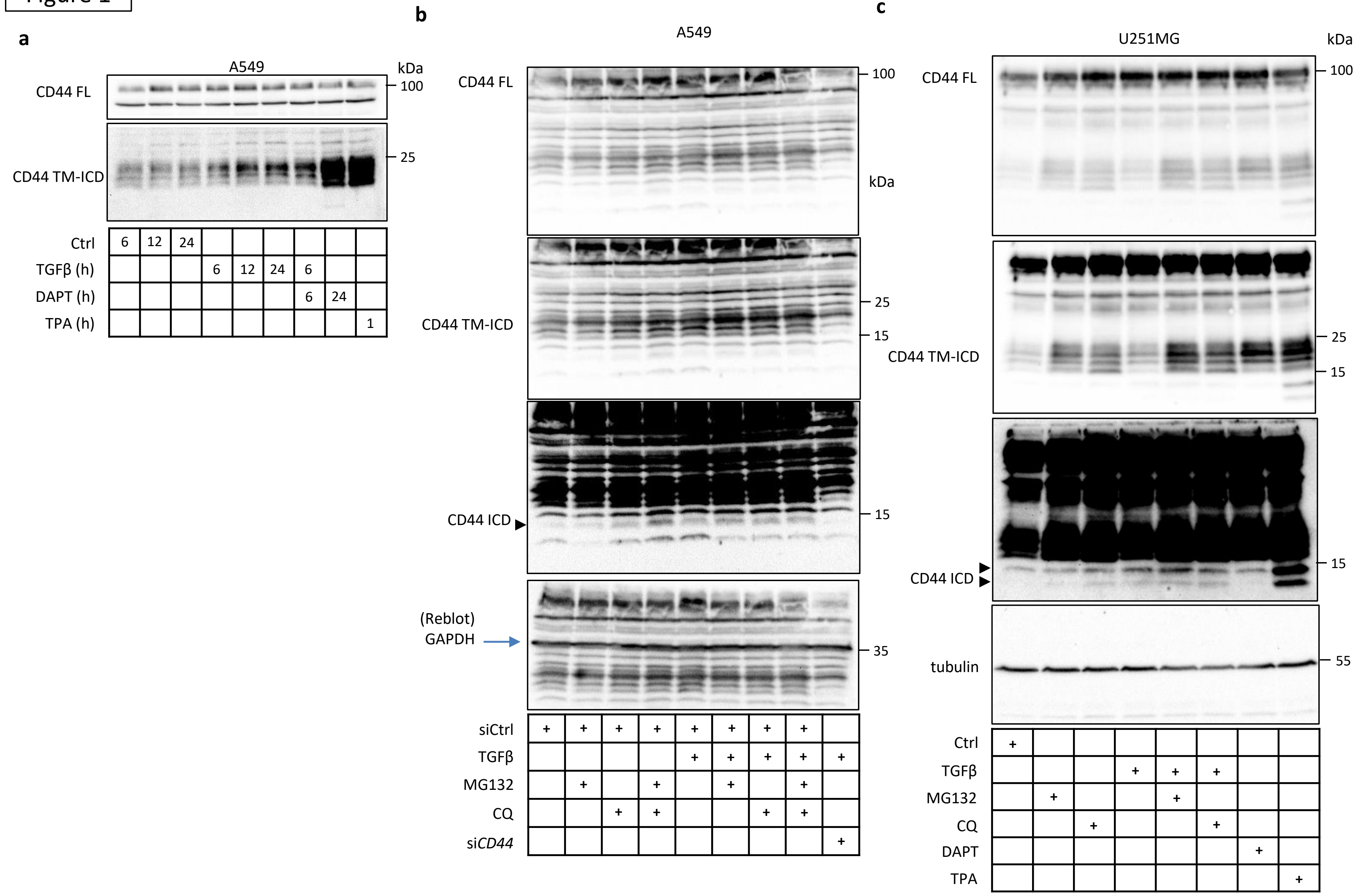
a
A549
kDa
CD44 FL
100
CD44 TM-ICD
25
Ctrl 6 12 24
TGFβ (h) 6 12 24 6
DAPT (h) 6 24
TPA (h) 1
b
A549
CD44 FL
100
kDa
CD44 TM-ICD
25
15
CD44 ICD
15
(Reblot)
GAPDH
35
siCtrl
TGFβ
MG132
CQ
siCD44
c
U251MG
kDa
CD44 FL
100
CD44 TM-ICD
25
15
CD44 ICD
15
tubulin
55
Ctrl
TGFβ
MG132
CQ
DAPT
TPA

Figure 2

e

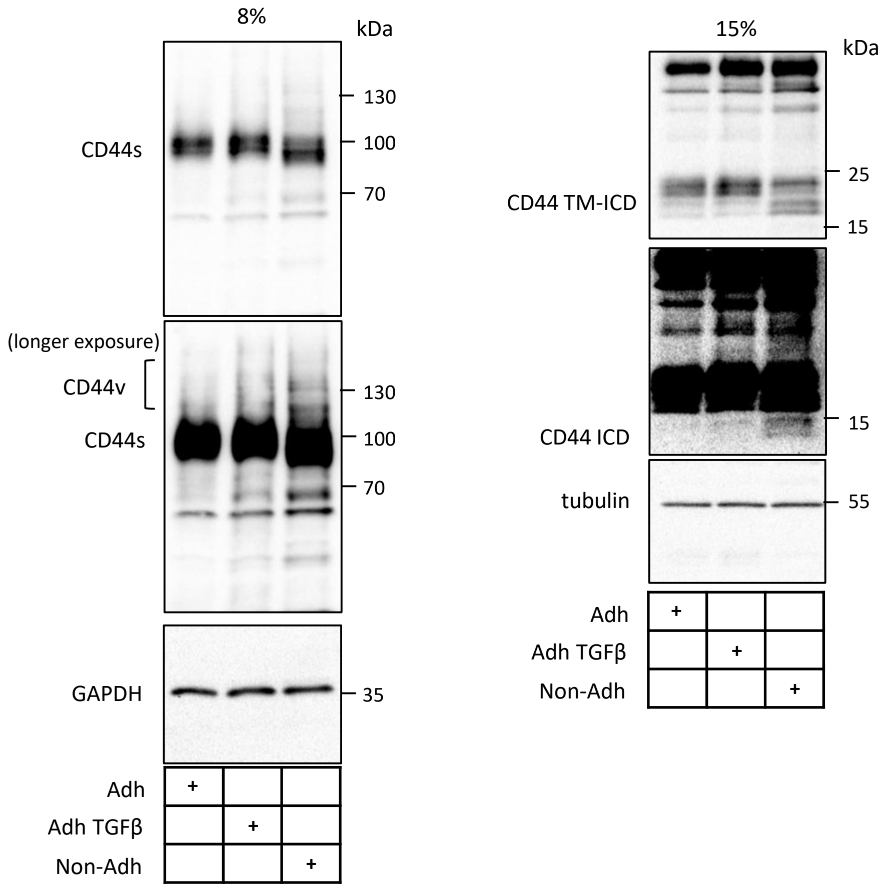
8%
kDa
130
CD44s
100
70
(longer exposure)
CD44v
130
CD44s
100
70
GAPDH
35
Adh
+
Adh TGFβ
+
Non-Adh
+
15%
kDa
25
CD44 TM-ICD
15
CD44 ICD
15
tubulin
55
Adh
+
Adh TGFβ
+
Non-Adh
+

Figure 3

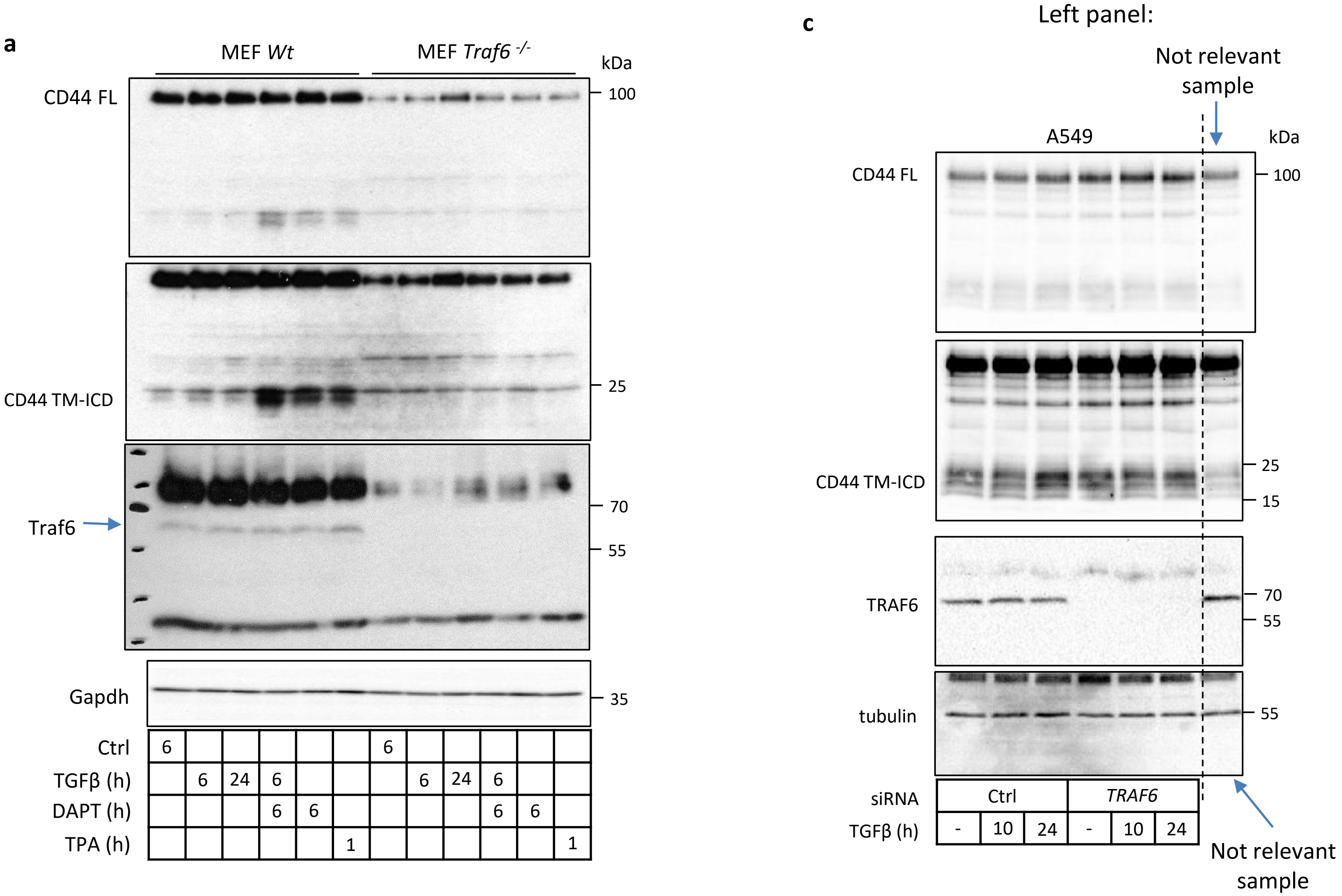

a
MEF Wt
MEF Traf6 -/-
kDa
CD44 FL
100
CD44 TM-ICD
25
Traf6
70
55
Gapdh
35
Ctrl 6 6
TGFβ (h) 6 24 6 6 24 6
DAPT (h) 6 6 6 6
TPA (h) 1 1
c
Left panel:
Not relevant sample
A549
kDa
CD44 FL
100
CD44 TM-ICD
25
15
TRAF6
70
55
tubulin
55
siRNA Ctrl TRAF6
TGFβ (h) - 10 24 - 10 24
Not relevant sample

Figure 3 continued

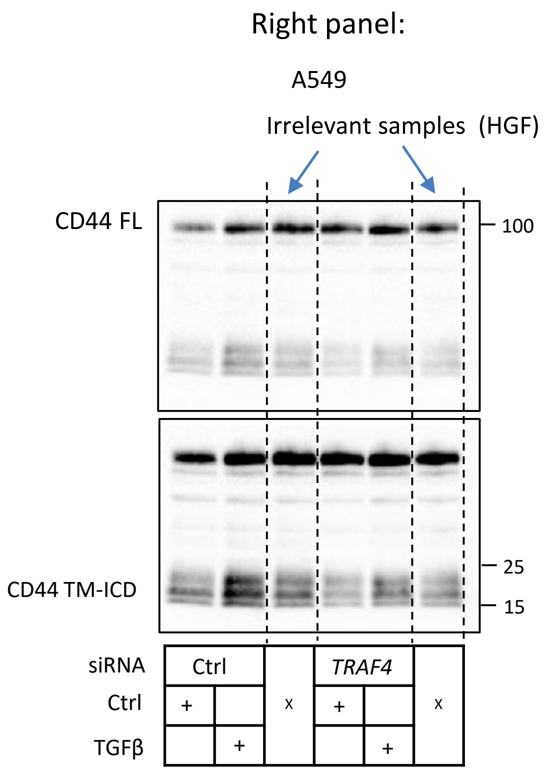

Figure 4

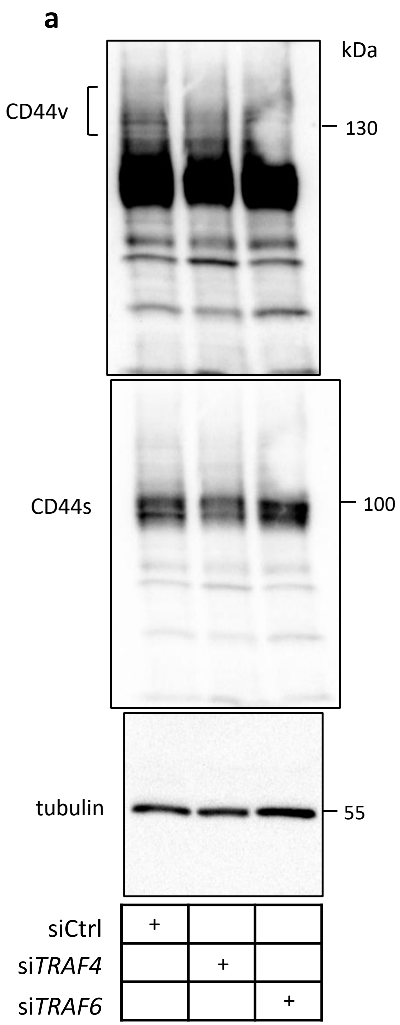

Figure 5

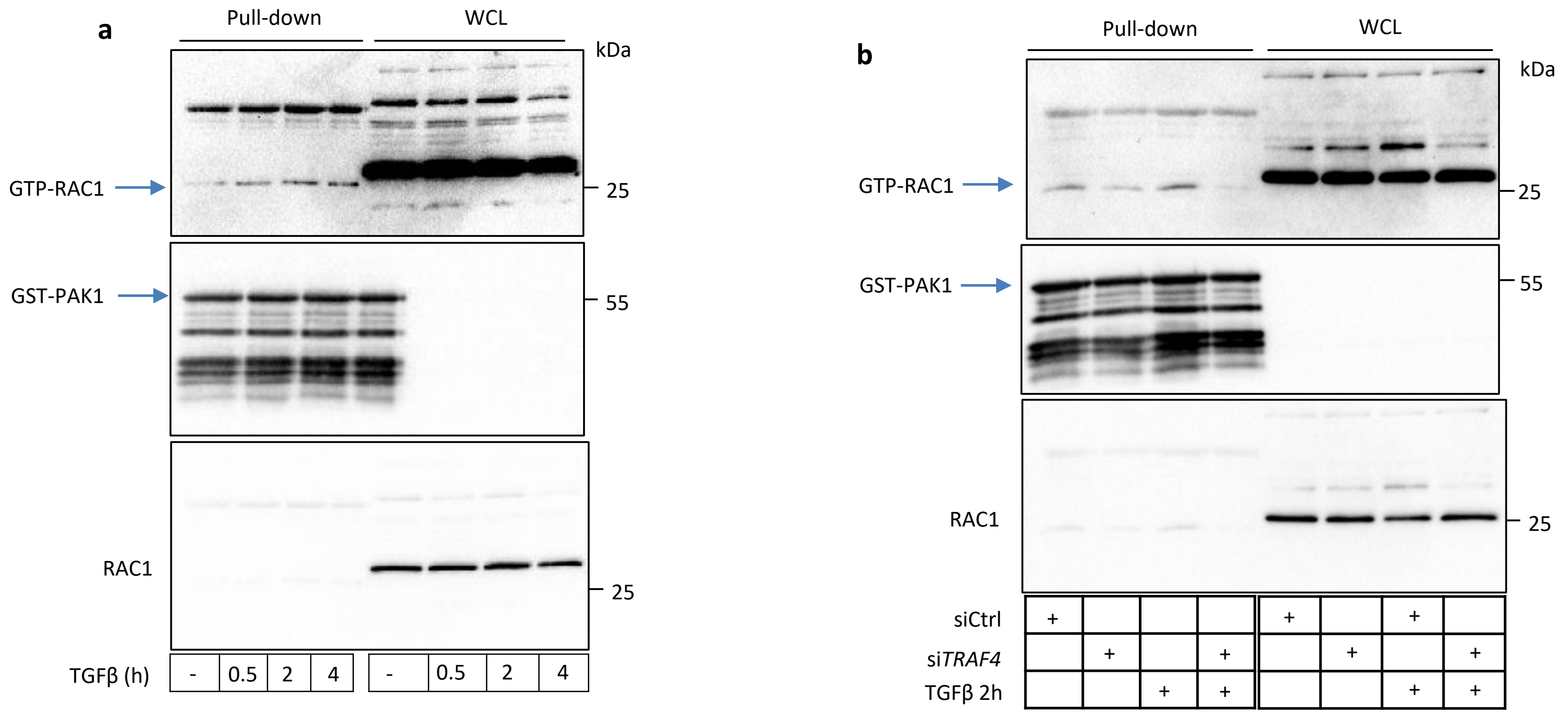
a
Pull-down
WCL
kDa
GTP-RAC1
25
GST-PAK1
55
RAC1
25
TGFβ (h)
-
0.5
2
4
-
0.5
2
4
b
Pull-down
WCL
kDa
GTP-RAC1
25
GST-PAK1
55
RAC1
25
siCtrl
siTRAF4
TGFβ 2h

Figure 5 continued

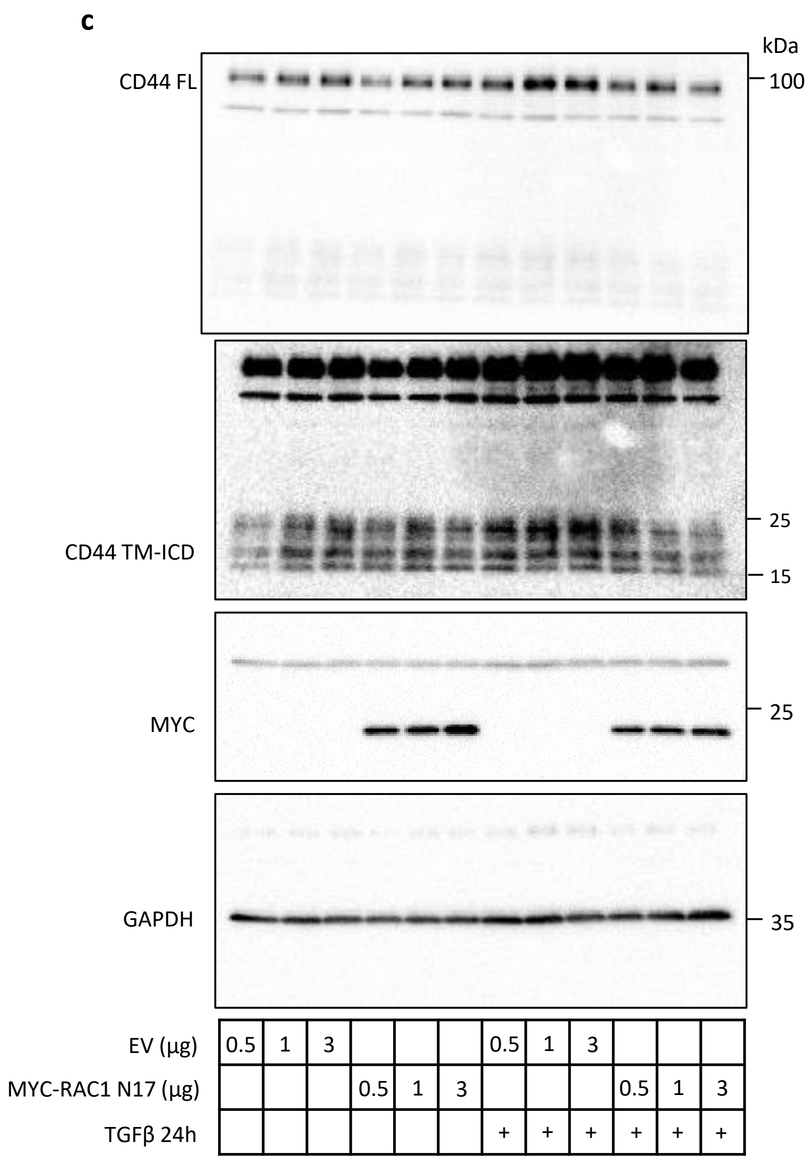

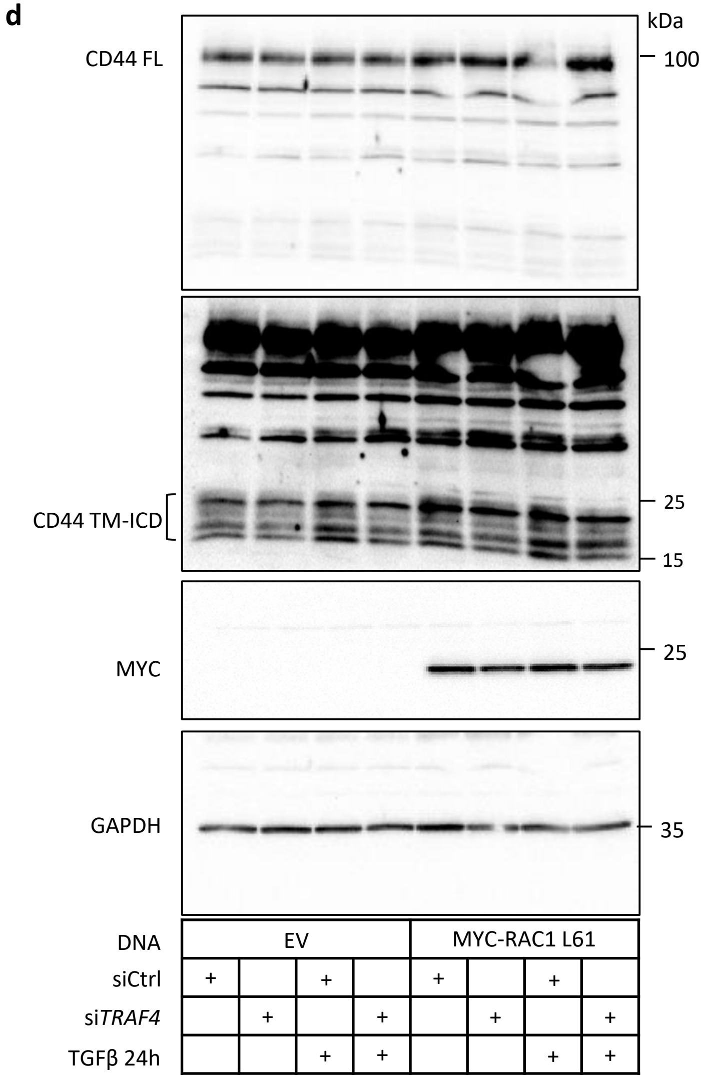

Figure S1

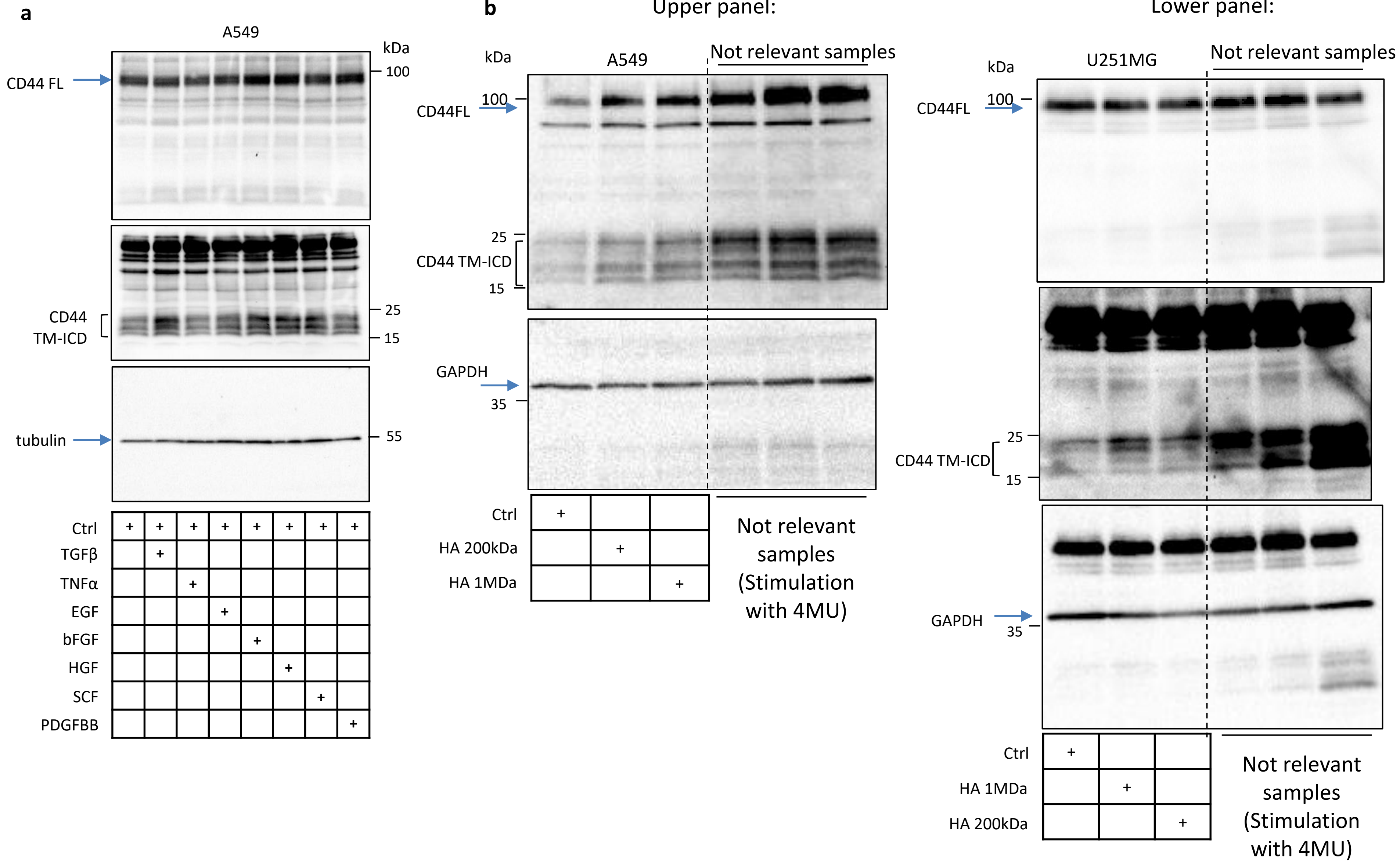
a
A549
kDa
CD44 FL
100
CD44
TM-ICD
25
15
tubulin
55
Ctrl
TGFβ
TNFα
EGF
bFGF
HGF
SCF
PDGFBB
b
Upper panel:
kDa
A549
Not relevant samples
CD44FL
100
25
CD44 TM-ICD
15
GAPDH
35
Ctrl
HA 200kDa
HA 1MDa
Not relevant samples (Stimulation with 4MU)
Lower panel:
kDa
U251MG
Not relevant samples
CD44FL
100
25
CD44 TM-ICD
15
GAPDH
35
Ctrl
HA 1MDa
HA 200kDa
Not relevant samples (Stimulation with 4MU)

Figure S1 continued

c

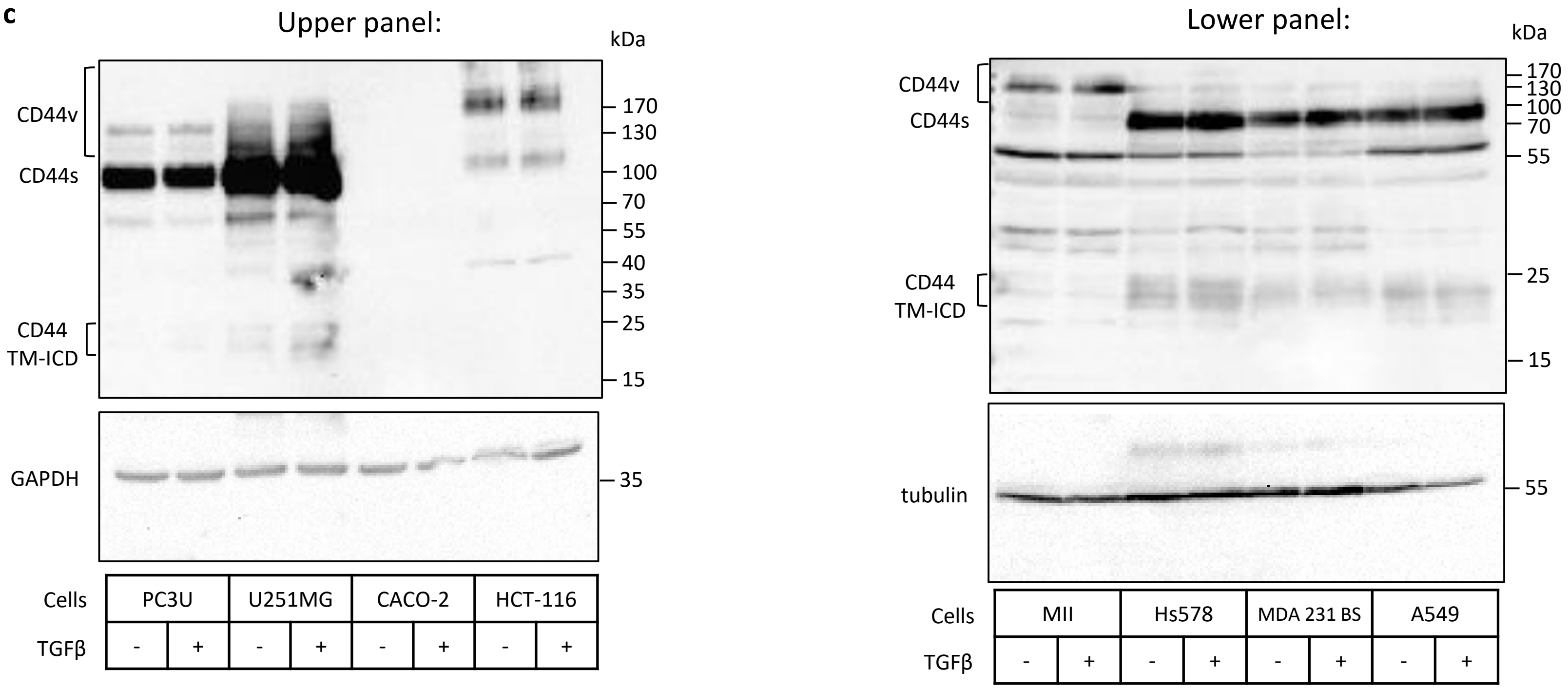

Figure S2

a

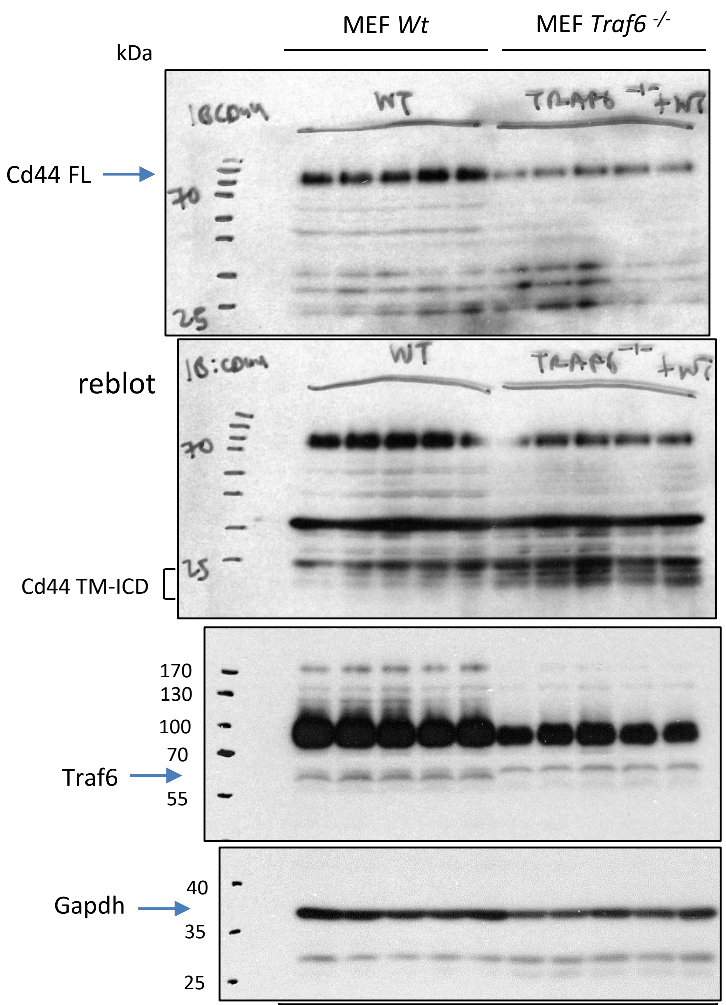

cDNA	Flag-Traf6 Wt									
Ctrl	6					6				
TGFβ (h)		6	24				6	24		
DAPT (h)				6					6	
TPA (h)					1					1

b

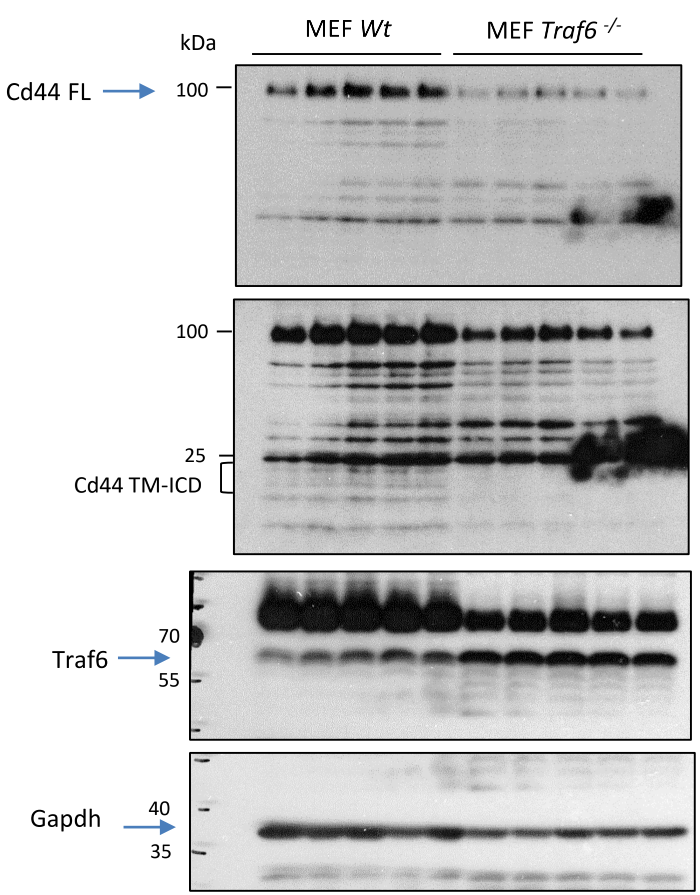

cDNA	Flag-Traf6 C70A									
Ctrl	6					6				
TGFβ (h)		6	24				6	24		
DAPT (h)				6					6	
TPA (h)					1					1
